# Constitutive PGC-1α Overexpression in Skeletal Muscle Does Not Contribute to Exercise-Induced Neurogenesis

**DOI:** 10.1007/s12035-020-02189-6

**Published:** 2020-11-16

**Authors:** Lars Karlsson, María Nazareth González-Alvarado, Reza Motalleb, Yafeng Wang, Yong Wang, Mats Börjesson, Changlian Zhu, Hans-Georg Kuhn

**Affiliations:** 1 Center for Brain Repair and Rehabilitation, Institute for Neuroscience and Physiology, University of Gothenburg, Gothenburg, Sweden; 2 The Queen Silvia Children’s Hospital, Sahlgrenska University Hospital, Region of Western Sweden, Gothenburg, Sweden; 3 Henan Key Laboratory of Child Brain Injury, Institute of Neuroscience and Third Affiliated Hospital of Zhengzhou University, Zhengzhou, China; 4 Department of Pediatrics, Children’s Hospital Affiliated to Zhengzhou University, Zhengzhou, China; 5 Department of Molecular and Clinical Medicine, Sahlgrenska Academy and Center for Health and Performance, University of Gothenburg, Gothenburg, Sweden; 6 Sahlgrenska University Hospital/Östra, Region of Western Sweden, Gothenburg, Sweden

**Keywords:** PGC-1α, Transgenic mice, Hippocampal neurogenesis, Voluntary running, Aging, Immunohistochemistry

## Abstract

Physical exercise can improve age-dependent decline in cognition, which in rodent is partly mediated by restoration of an age-dependent decline in neurogenesis. Exercise-inducible myokines in the circulation present a link in muscle-brain crosstalk. The transcription factor PGC-1α regulates the release of such myokines with neurotrophic properties into the circulation. We study how chronic muscular overexpression of PGC-1α could contribute to exercise-induced effects on hippocampal neurogenesis and if this effect could be enhanced in a running wheel paradigm. We used 3- and 11-month-old transgenic mice with overexpression of PGC-1α under the control of muscle creatinine kinase promoter (MCK-PGC-1α), which have a constitutively developed endurance muscle phenotype. Wild-type and MCK-PGC-1α mice were single housed with free access to running wheels. Four weeks of running in female animals increased the levels of newborn cells, immature neurons, and, for young animals, new mature neurons, compared to sedentary controls. However, no difference in these parameters was observed between wild-type and transgenic mice under sedentary or running conditions. Multiplex analysis of serum cytokines, chemokines, and myokines suggested several differences in serum protein concentrations between genotypes with musclin found to be significantly upregulated 4-fold in male MCK-PGC-1α animals. We conclude that constitutive muscular overexpression of PGC-1α, despite systemic changes and difference in serum composition, does not translate into exercise-induced effects on hippocampal neurogenesis, independent of the age of the animal. This suggests that chronic activation of PGC-1α in skeletal muscle is by itself not sufficient to mimic exercise-induced effects or to prevent decline of neurogenesis in aging.

**Supplementary Information:**

The online version contains supplementary material available at 10.1007/s12035-020-02189-6.

## Introduction

Physical exercise, especially aerobic exercise, can prevent and treat health conditions in many organs in the body. For example, exercise improves cardiovascular health, bone mineral density, decreases risk for cancer, stroke, diabetes, and many other illnesses. Exercise also has neuroprotective effects on the brain with aerobic exercise being associated with improved cognition, neuronal function, and structure of the brain [1, 2]. Exercise enhances neurogenesis, synaptic plasticity, and angiogenesis in the dentate gyrus of the hippocampus [3], in part mediated by an exercise-induced increase in neurotrophic factors in the brain. However, it is still unclear how exercise can exert effects on the central nervous system (CNS), particularly through signaling by factors in the circulation [4]. Growth factors such as brain-derived neurotrophic factor (BDNF) [5], vascular endothelial growth factor (VEGF) [6], and insulin-like growth factor-1 (IGF-1) [7] are essential for exercise-induced neurogenesis, but other circulating factors may also regulate this process. Blood vessels in the vicinity of the hippocampal neurogenic niche can influence neural stem cell proliferation and differentiation through secretion of factors from endothelial cells and by allowing passage of factors across the blood–brain barrier [8], indicating that hippocampal neural stem and progenitor cells can readily respond to changes in oxygen, nutrients, metabolites, hormones, and other factors in the bloodstream. Skeletal muscle, adipose tissue, and liver secrete various molecules and vesicles into the circulation during exercise with systemic effects on metabolism [9]. The effects of these secreted molecules have been thoroughly studied in peripheral tissues, but the effects of exercise-induced metabolic changes on structural and functional changes in the CNS as well as underlying signaling mechanisms are yet to be elucidated.

The transcription factor peroxisome proliferator-activated receptor gamma co-activator 1-alpha (PGC-1α) is a master regulator of mitochondrial biogenesis, a process enhancing mitochondrial function and oxidative capacity in cells [10]. PGC-1α is induced and activated in skeletal muscle from acute exercise and regular exercise training, considered to be a key driver of exercise-induced effects in skeletal muscle, including the release of neurotrophic factors into the circulation [11–13]. PGC-1α is induced and activated by mitogen-activated protein kinase (MAPK) p38 and adenosine monophosphate-activated protein kinase (AMPK) during exercise [14]. PGC-1α induces endurance training adaptations in skeletal muscle through co-activation of several nuclear receptors and other transcription factors [15]. PGC-1α mediates cellular adaptation to exercise that includes enhanced mitochondrial biogenesis, oxidative capacity, lipoperoxidation, glucose uptake, angiogenesis, and muscle fiber-type switching [12, 16]. PGC-1α also mediates exercise-induced secretion of factors into the circulation with neurotrophic potential. For example, one of the downstream factors of PGC-1α is fibronectin type III domain-containing protein 5 (FNDC5), which is cleaved and released into the circulation as irisin [17]. The peripheral expression of this protein results in upregulated hippocampal BDNF levels [17] and improvement in synaptic plasticity in a model of Alzheimer’s disease [18]. FNDC5, along with other muscle-derived exercise factors, such as cathepsin B [19] and beta-hydroxybutyrate [20], constitutes a direct molecular signaling from muscle to brain [21].

Transgenic mice with constitutive skeletal muscle-specific PGC-1α overexpression under the muscle creatinine kinase promoter (MCK-PGC-1α) have a genetically enhanced endurance exercise phenotype [16]. These transgenic animals are protected from denervation and disuse-induced muscle atrophy [22, 23], as well as age-related motor dysfunction [24, 25]. On a systemic level, MCK-PGC-1α mice show improvement in whole body metabolism with aging [25], enhanced improvement in glucose homeostasis with exercise [26], and are protected from stress-induced neuroinflammation [27]. In a study by Peng and colleagues, MCK-PGC-1a mice were reported to have improved kidney energy metabolism with protection against kidney damage and fibrosis, an effect that was mediated by circulating irisin [28]. Exercise can ameliorate age-dependent decline in neurogenesis [29] through protection of the hippocampal stem cell pool with aging and sustained neuronal lineage commitment of neural stem and progenitor cells [30]. Endurance exercise induces PGC-1α-dependent mitochondrial recovery in a mouse model of accelerated aging that recapitulates many features of human aging [31, 32], a model in which muscle-specific overexpression of PGC-1α is able to ameliorate both age-related mitochondrial dysfunction in muscle, as well as age-related signs of anemia [33].

We hypothesize that skeletal muscle activation via PGC-1α overexpression releases exercise-inducible molecules into the circulation which contribute to exercise-induced neurogenesis. To determine if chronic PGC-1α activation in skeletal muscle could prevent age-dependent decline in neurogenesis or enhance levels of exercise-induced neurogenesis in aging, we study hippocampal neurogenesis in young and middle-aged MCK-PGC-1α female mice subjected to a running wheel paradigm.

## Material and Methods

### Animals

Transgenic MCK-PGC-1α animals on C57BL/6J background (The Jackson Laboratory, Stock no. 008231), a kind gift from Dr. Bruce Spiegelman (Harvard Medical School, Boston, MA), have been previously described [16]. Female C57BL/6J (Charles River, Germany) mice were used for breeding purposes. Animals were housed at a constant temperature (24 °C) with 50–60% relative humidity. A 12-h light/dark cycle was maintained with lights from 07:00 to 19:00 hours and with ad libitum access to food and water. All experiments were approved by the Gothenburg Ethical Committee on Animal Research (#317-2012 and #181-2015) and performed in accordance with relevant guidelines and regulations. Transgenic animals were bred as hemizygous mice and wild-type (C57BL/6J) littermates were used as controls. Prior to inclusion in experiments, females were group housed with age-matched females, and males were group housed with male littermates.

### Genotyping

Genotyping was performed as described previously [34]. Primers targeting interleukin 2 (internal control; forward, (5′-CTAGGCCACAGAATTGAAAGATCT-3′; reverse, 5′-GTAGGTGGAAATTCTAGCATCATCC-3′) and the PGC-1α transgene insert (forward, 5′-AGCCGTGACCACTGACAACGAG-3′; reverse, 5′-GCTGCATGGTTCTGAGTGCTAAG-3′) were used for genotyping.

### BrdU Labeling and Voluntary Running Procedure

Female and male wild-type and MCK-PGC-1α mice at 2 and 10 months of age were single housed in cages with free access to running wheels (ENV-047, Med Associates, Fairfax, VT, USA). After 5 days of acclimatization, half of the running wheels were unlocked and animals were given daily intraperitoneal injections of BrdU (50 mg/kg) for 5 consecutive days. Throughout the experiment running wheel activity was recorded wirelessly. Wild-type (WT) and transgenic (TG) animals were randomized to voluntary running or as sedentary controls. For 3-month-old animals, 34 females (6 WT sedentary, 10 WT runners, 10 TG sedentary, and 8 TG runners) and 18 males (9 WT runners and 9 TG runners) were included in the experiment. For 11-month-old animals, 32 females (7 WT sedentary, 7 WT runners, 9 TG sedentary, and 9 TG runners; only the first week of running activity was monitored for 2 WT runners and 2 TG runners) and 34 males (9 WT sedentary, 9 WT runners, 7 TG sedentary, and 9 TG runners; only the first week of running activity was monitored for 1 WT runners and 3 TG runners) were included in the experiment. Animals were euthanized and perfused 28 days after the first day of BrdU injection for immunohistochemical analysis of cytogenesis and neurogenesis.

### Tissue Processing

Four weeks following voluntary exercise, mice were deeply anesthetized with a peritoneal injection of 50 mg/kg sodium thiopental during the inactive phase of the animals. Blood was extracted through cardiac puncture using a 27-gauge needle, which was allowed to coagulate in a low protein-binding microcentrifuge tube (Maxymum Recovery, Corning Life Sciences, Corning, NY, USA) for 1 h. After centrifugation at 3000*g* for 10 min, serum was transferred into a new low protein-binding tube, frozen on dry ice, and stored at − 80 °C until further use. The gastrocnemius muscle was dissected and flash frozen in isopentane containing dry ice, and stored at − 80 °C until further use. Animals were transcardially perfused with cold saline solution (0.9% NaCl) followed by 4% paraformaldehyde (PFA) in phosphate-buffered solution (PBS). The brains were immersion-fixed in PFA and subsequently cryoprotected in 30% sucrose in 0.1 M PBS after 24 h. Left hemispheres were sectioned sagittally at 25 μm thickness and collected in series of 12 sections for immunohistochemistry, using a sliding microtome (SM2000R, Leica Microsystems, Wetzlar, Germany), modified for frozen sectioning. Sections were stored at 4 °C in cryoprotectant solution (TCS) containing 25% glycerol and 25% ethylene glycol in 0.1 M PBS.

### Immunohistochemistry

Free-floating sections were rinsed in Tris-buffered saline (TBS). An antigen retrieval step in 10 Mm sodium citrate (pH 6) for 20 min at 80 °C, with a 10-min cooling step, was performed before immunohistochemistry. For 3,3′-diaminobenzidine (DAB) immunohistochemical detection of doublecortin (DCX), sections were incubated in 0.6% H_2_O_2_/TBS for 30 min at room temperature (RT). For BrdU/NeuN double staining, sections were incubated in 2 N HCl for 30 min at 37 °C and neutralized in 0.1 M borate buffer (pH 8.5) for 10 min at RT. For all immunostainings, sections were incubated in blocking solution containing 0.1% Triton X-100, 3% donkey serum (Jackson ImmunoResearch Laboratories, Baltimore, PA, USA) in TBS for 30 min at RT. Primary antibodies were diluted in blocking solution and incubated at 4 °C for 3 days in goat anti-DCX 1:250 (Santa Cruz Biotechnology, Cat# sc-8066, RRID: AB_2088494) or 2 days in rat anti-BrdU 1:500 (Bio-Rad, Cat# OBT0030, RRID: AB_609568) and mouse anti-NeuN 1:2000 (Millipore, Cat# MAB377, RRID: AB_2298772). After rinsing in TBS, sections were incubated at RT for either 1 h (biotinylated) or 2 h (fluorescence) with secondary antibody in blocking solution as follows: with biotinylated donkey anti-goat 1:2000 (Jackson ImmunoResearch Labs, Cat# 705-065-147, RRID: AB_2340397) for DCX-DAB and with donkey anti-mouse 555 IgG 1:1000 (Thermo Fisher Scientific, Cat# A-21202, RRID: AB_141607) and donkey anti-rat 488 IgG 1:1000 (Thermo Fisher Scientific, Cat# A-21208, RRID: AB_2535794) for BrdU/NeuN immunofluorescence. DAB sections were washed and incubated in avidin–biotin–peroxidase (Vector Laboratories, Cat# PK-7100, RRID: AB_2336827) for 1 h and then rinsed and incubated 5–10 min in DAB (DAB Safe, Saveen Werner, Limhamn, Sweden) solution containing 0.25 mg DAB, 0.3 μl 30% H_2_O_2_, and 5 μl 8% NiCl_2_ per 1 ml TBS. Finally, the sections were rinsed in tap water and TBS and mounted from 0.1 M PBS on glass slides using X-TRA-kitt® (Medite, Redmondstown, Ireland) for coverslipping. For immunofluorescence, sections were rinsed and mounted from 0.1 M PBS onto glass slides. After allowing sections to dry, glass slides were submerged in 1% Sudan Black in EtOH for 5 min and rinsed thoroughly in PBS. Finally, sections were coverslipped using prolong Gold with DAPI (Molecular Probes, Eugene, OR, USA).

The following numbers of animals were stained and quantified for analysis of BrdU^+^ and BrdU^+^/NeuN^+^ cells in 3-month-old female animals, 5 WT sedentary, 8 WT runners, 9 TG sedentary, and 7 TG runners (1 WT sedentary, 2 WT runners, 1 TG sedentary, and 1 TG runner were excluded from analysis due to insufficient numbers of sections after mounting) and in 11-month-old female animals, 7 WT sedentary, 7 WT runners, 9 TG sedentary, and 9 TG runners. For analysis of DCX^+^ cells in 3-month-old female animals, 6 WT sedentary, 7 WT runners, 10 TG sedentary, and 8 TG runners (3 WT runners were excluded from analysis due to insufficient numbers of sections after mounting) and in 11-month-old female animals, 5 WT sedentary, 5 WT runners, 7 TG sedentary, and 10 TG runners were stained and quantified (2 WT sedentary, 2 WT runners, and 2 TG sedentary were not included in immunohistochemistry). For analysis of DCX+ cells in 11-month-old male animals, 9 WT sedentary, 10 WT runners, 7 TG sedentary, and 10 TG runners were stained and quantified (1 WT runner and 1 TG runner were excluded from analysis due to insufficient number of sections after mounting).

### Imaging and Quantification

Investigator-blinded stereological quantification was done with a Leica DM6000B microscope and software (StereoInvestigator v10.40, MBF Bioscience). BrdU- and DCX-positive cells were quantified at 40× optical magnification. For volumetric analysis of the granule cell layer (GCL), the area of the GCL was quantified by tracing the borders of the layer at 20× optical magnification and multiplied by the thickness of the section. For all stereological quantification, in a mediolateral direction, quantification started on the first sagittal section were the dorsal and ventral dentate gyrus no longer were interconnected, and ended with the first sagittal section were the dorsal blade of the GCL no longer could be distinguished from the ventral blade. For stereological analysis, data from lost or damaged sections were estimated as the average value of the two anatomically adjacent sections. For analysis of NeuN/BrdU co-labeling a Leica SP2 confocal microscope was used. Co-localization was determined at 20× optical magnification at close to 1 airy-units (AU) with 2.5× digital zoom and a sequential scanning mode. The NeuN antibody has a limited penetration into free-floating sections, allowing only phenotyping the BrdU^+^ cells at the surfaces of the section where the NeuN labeling was homogenous and sufficiently strong. Approximately half of the BrdU^+^ cells were phenotyped for each animal. Images have been processed with Photoshop (Adobe, Photoshop CC 2017) for publication. All images were cropped and adjusted for brightness, and confocal images were level adjusted for enhanced signal-to-noise ratio per channel.

### Immunoblotting

Gastrocnemius muscle from 9- to 12-month-old WT and TG animals was harvested as described above under the section “Tissue processing.” Muscle specimens were homogenized by sonication (Branson, Sonifier 250) in ice-cold RIPA buffer containing 150 mM sodium chloride, 1.0% Triton X-100, 0.5% sodium doxycholate, 0.1% sodium dodecyl sulfate, and 50 mM Tris (pH 8), with protease inhibitor cocktail (Thermo Fisher Scientific, 87786). Protein concentration was determined using the bicinchoninic acid method and equal amounts of denatured protein were loaded on NuPAGE 4–12% Bis-Tris gels (Invitrogen). Samples were run on gels and transferred to reinforced nitrocellulose membranes (Bio-Rad). After blocking with 5% bovine serum albumin (BSA) in TBST buffer (20 mM Tris, 150 mM NaCl, and 0.1% Tween 20, pH 7.6) for 30 min at room temperature, membranes were incubated with the primary antibodies rabbit-anti-PGC1a 1:1000 (Abcam, Cat# ab54481, RRID: AB_881987) overnight in TBST with 3% BSA at 4 °C. After washing, membranes were incubated with peroxidase-labeled goat anti-rabbit IgG antibody 1:2000 (Vector Laboratories, Cat# PI-1000, RRID: AB_2336198). Total protein was detected by using Ruby Protein Blot Stain (Thermo Fisher Scientific, S11719). Immunoreactive species were visualized with the SuperSignal West Pico PLUS Chemiluminescent Substrate (Thermo Fisher Scientific, 34580) using a LAS-3000 CCD camera (Fujifilm, Japan). Protein band densities were normalized to total protein in entire well lanes. Due to the fact that western blot sample preparation occurred at different time points for sedentary and voluntary running animals (see full blot in Online Resource Supplementary Figure S1A), we refrain from making direct statistical comparison between these two groups.

### Multiplex Protein Analysis

Serum from single-housed 11-month-old males was thawed and applied to multiplex assays for detection of serum chemokines, cytokines (Thermo Fisher Scientific, Cat# EPX360-26092-901, RRID: AB_2576123) and myokines (Milliplex, MMYOMAG-74K, Merck, Kenilworth, NJ, USA). Multiplex microplates were analyzed using a Bio-Plex 100 system (Bio-Rad) according to manufacturer’s instructions. See full list of analytes in Online Resource Supplementary Table S1. Concentrations were determined based on the average of technical duplicates and wells with bead count of less than 20 were excluded to ensure high precision. In cases where the concentrations were below the detection range, concentration value was set as midpoint between 0 and minimum detection limit of the analysis. In the multiplex protein analysis data of the study, sedentary animals from a previous study have been included as data points in the graphs for baseline purpose [35].

### Statistical Analysis

Data were processed and analyzed using Microsoft Excel 2017 (Microsoft Corp., RRID: SCR_016137) and GraphPad Prism 7 (RRID: SCR_002798). Appropriate tests were selected, as specified in the text, based on normality and homogeneity of variance. Normality was determined by visual inspection of density plotted logged and unlogged data. For data adhering to normality and equality of variances, two-way analysis of variance (ANOVA) was used. For running data, two-way repeated-measures ANOVA (RM-ANOVA) was used and matching was effective for all tests. Three-way ANOVA was performed in SPSS Statistics v24 (IBM Corp., RRID: SCR_002865). For non-parametric data, the Scheirer–Ray–Hare extension of the Kruskal–Wallis test [36] was used as a non-parametric equivalent of the two-way ANOVA. Running distances were analyzed against protein concentrations in wild-type and transgenic mice using either linear regression for data adhering to normality, or Kendall rank correlation [37] for both parametric and non-parametric data. Linear correlation coefficient and *p* values were calculated with Pearson statistics using the limma package [38] in R (University of New Zealand, Auckland, New Zealand). False discovery rate (FDR) was calculated with the Benjamini–Hochberg method [39] using an online calculator [40]. Kendall rank correlation was used to calculate ranking correlation between running distance and cytokine concentrations in wild-type and transgenic mice. Kendall’s tau coefficient and corresponding *p* values and FDR-adjusted *p* values were calculated based on non-parametric tau statistics in R. Significance level was set to 0.05 for all experiments. Boxplots are plotted with whiskers and outliers using the Tukey method. Boxes are plotted with the 25th percentile, the median, and the 75th percentile. Whiskers are plotted as the minimum and maximum values, unless in the case of outliers. If a value is either greater than the sum of the 75th percentile and the interquartile range multiplied by 1.5, or less then the 25th percentile minus the interquartile range multiplied by 1.5, it is plotted as an individual point (outlier). The G*Power software (version 3.1.9.7) has been used for post hoc power calculation of multiplex analysis using the Bonferroni correction [41].

## Results

**Fig. 1:**
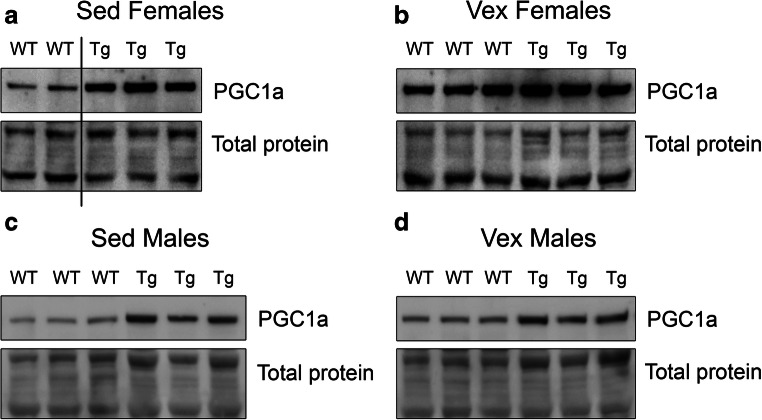
Overexpression of PGC-1α in skeletal muscle leads to increased PGC-1α protein levels in skeletal muscle. Images shows western blot against on gastrocnemius homogenate from wild-type and transgenic MCK-PGC-1α animals for **a** 7–10-month-old female sedentary animals (line indicates a cropped out lane of a male animal, see full blot below), **b** female running animals, **c** 7-month-old male sedentary animals, and **d** male running animals. Immunoblotting against PGC-1α (91 kDa) and total protein. See Online Resource Supplementary Figure S1 for full immunoblot of female animals and Supplementary Figure S2 for full immunoblot of male animals. WT, wild type; Tg, transgenic; Sed, sedentary; Vex, voluntary exercise

Mice that overexpress PGC-1α in skeletal muscle under the muscle creatinine kinase promoter [16] were used to investigate if animals with an endurance muscle phenotype would display different exercise-induced effects on neurogenesis. In particular, we studied effects of exercise, aging and genotype on hippocampal neurogenesis. The MCK-PGC-1α mouse model used in this study has previously been validated by us to have a developed endurance muscle phenotype [34, 35]. Western blot on gastrocnemius homogenate of middle-aged (7–10-month-old) sedentary animals show that MCK-PGC-1α animals have around 4-fold increased PGC-1α levels in comparison to wild-type animals (*t* test with Welch correction, *n* = 3, *p* = 0.043; all female animals except for one male individual in WT sedentary; see Fig. 1 and Online Resource Supplementary Figure S1), whereas no difference was detected in gastrocnemius PGC-1α protein levels between genotypes after 4 weeks of voluntary running (*t* test with Welch correction, *n* = 3, *p* = 0.17). Due to a difference in total protein stain for sedentary and voluntary running animals (Online Resource Supplementary Figure S1A), we refrain from making statistical comparison between these two groups. Running females in this experiment had an average daily running activity of 4.1 ± 0.3 km/day. Western blot on gastrocnemius homogenate from 7-month-old male transgenic animals show around 3-fold increase in comparison to wild-type animals (two-way ANOVA; genotype effect, *p* < 0.0001; *n* = 3), whereas no difference was detected between sedentary and running animals (two-way ANOVA; running effect, *p* = 0.26; *n* = 3; all interactions being non-significant; Online Resource Supplementary Figure S2). Running males in this experiment ran for 4 weeks before sacrifice and had an average daily running activity of 0.4 ± 0.1 km/day.

### Skeletal Muscle PGC-1α Overexpression and Running-Induced Neurogenesis

We investigated potential protective and regenerative effects of chronic muscular overexpression of PGC-1α in exercise and aging. Wild-type and transgenic female animals were single housed and subjected to voluntary running at 2 and 10 months of age. Starting on day 1 of running, BrdU (50 mg/kg i.p.) was administered for 5 consecutive days to label newly generated cells. Animals were kept under running or sedentary control conditions until sacrifice for analysis 4 weeks later.

**Fig. 2:**
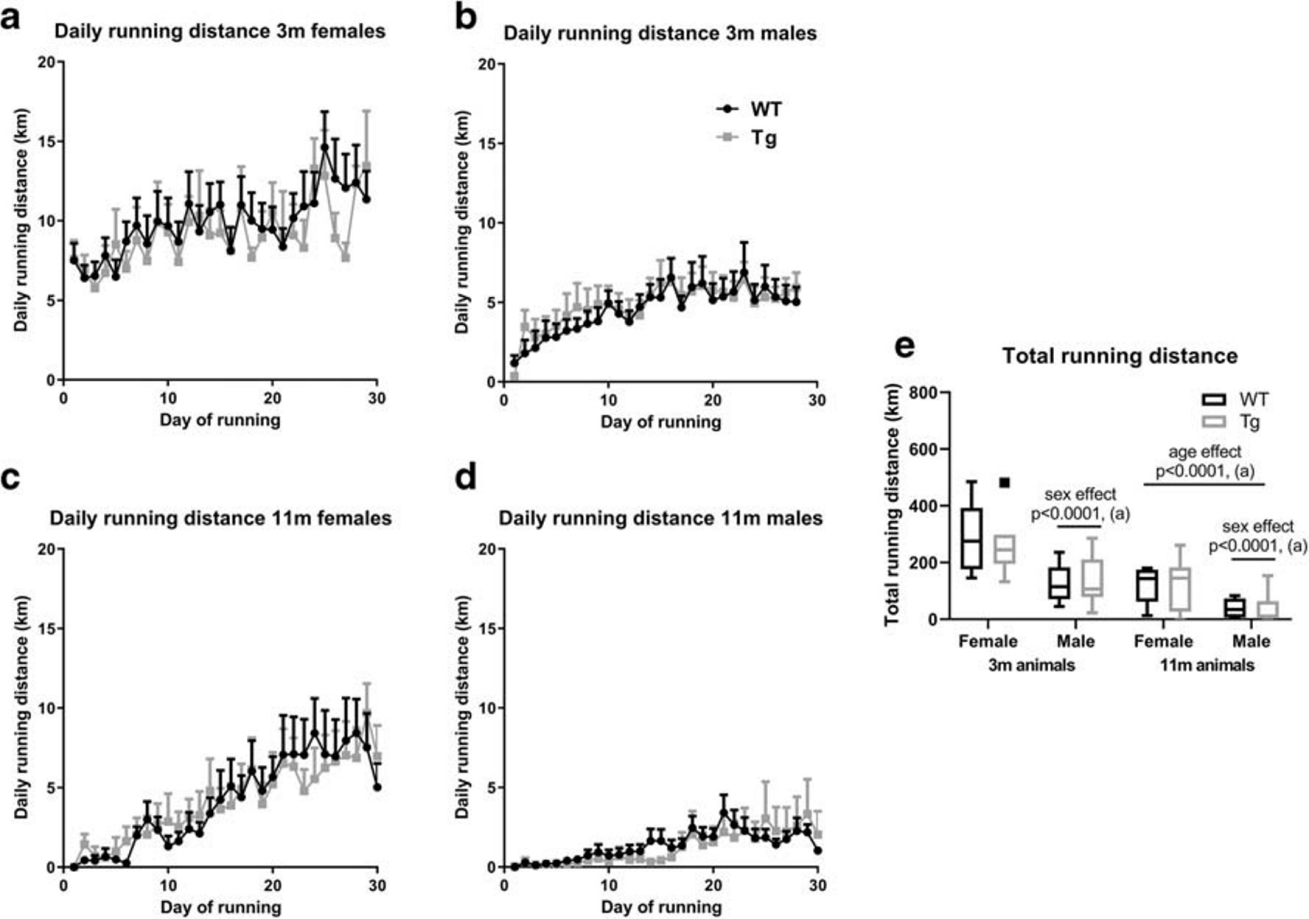
Daily running distances for 3- and 11-month-old wild-type and transgenic animals. Graphs show daily running distance for **a** 3-month-old females (*n* = 8–10), **b** 3-month-old males (*n* = 9), **c** 11-month-old females (*n* = 5–7), **d** 11-month-old males (*n* = 6–8), and **e** total running distances per age, sex, and genotype. Data in line graphs expressed as mean ± SEM. Data in box plot presented using the Tukey method. (a) (Three-way ANOVA; n = 5–10). WT, wild type; Tg, transgenic

Average daily running distance was similar for both young female wild-type (9.8 ± 1.9 km/day) and transgenic (9.1 ± 2.0 km/day) animals and was reduced in middle-aged female wild-type (4.1 ± 2.8 km/day) and transgenic (4.1 ± 2.4 km/day) animals (see Fig. 2). Compared to females, average daily running distance was considerably lower in young male wild-type (4.5 ± 1.5 km/day) and transgenic (4.8 ± 1.3 km/day) animals and was further decreased in middle-aged male wild-type (1.7 ± 1.1 km/day) and transgenic (1.2 ± 1.0 km/day) animals (see Fig. 2a–d). Average daily running distance was analyzed using a three-way ANOVA (*n* = 5–10) which showed significant main effects for age (*F*(1,63) = 36.2, *p* < 0.0001) and sex (*F*(1,63) = 24.5, *p* < 0.0001), but not for genotype (*F*(1,63) = 0.01, *p* = 0.92), with all interaction effects being non-significant. Total daily running distance was analyzed using a three-way ANOVA (*n* = 5–10) which showed significant main effects for age (*F*(1,63) = 31.5, *p* < 0.0001) and sex (*F*(1,63) = 27.8, *p* < 0.0001), but not for genotype (*F*(1,63) = 0.05, *p* = 0.82), with all interaction effects being non-significant (see Fig. 2e). Using two-way RM-ANOVA, no difference in average daily running distance could be detected between genotypes for 3-month-old females, 3-month-old males, 11-month-old females, or 11-month-old males (see Table 1).

**Table 1:** Genotype effect of two-way RM-ANOVA on daily average running distance

Analysis^a^	Genotype effect	Time effect	Interaction effect
3-month-old females^b^	0.13 (*p* = 0.72)	0.74 (*p* < 0.0001)	3.80 (*p* = 0.84)
3-month-old males^c^	0.06 (*p* = 0.82)	9.2 (*p* < 0.0001)	0.52 (*p* = 0.98)
11-month-old females^d^	<0.001 (*p* = 0.99)	10.2 (*p* < 0.0001)	0.56 (*p* = 0.97)
11-month-old males^e^	0.03 (*p* = 0.87)	6.0 (*p* < 0.0001)	0.78 (*p* = 0.79)

^a^See Fig. 2

^b^Data presented as test value of *F*(1,16) for genotype, *F*(28,448) for time and interaction, and *F*(16,448) for matching, along with the corresponding *p* value in parenthesis (*n* = 8–10)

^c^Data presented as test value of *F*(1,16) for genotype, *F*(27,432) for time and interaction, and *F*(16,432) for matching, along with the corresponding *p* value in parenthesis (*n* = 9)

^d^Data presented as test value of *F*(1,10) for genotype, *F*(29,290) for time and interaction, and *F(*10,290) for matching, along with the corresponding *p* value in parenthesis (*n* = 5–7)

^e^Data presented as test value of *F*(1,12) for genotype, *F*(29,348) for time and interaction, and *F*(12,348) for matching, along with the corresponding *p* value in parenthesis (*n* = 6–8)

**Fig. 3:**
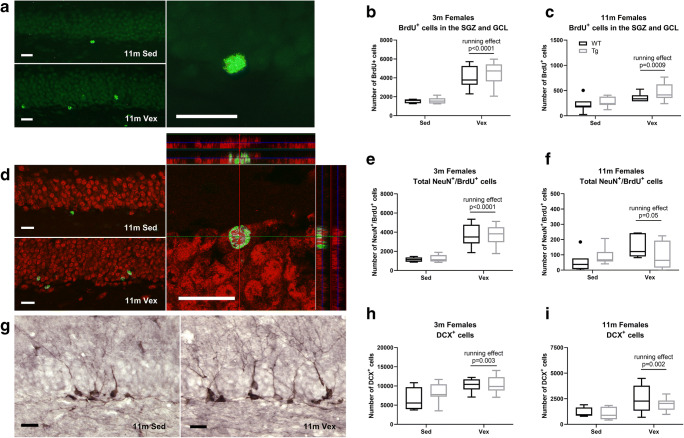
Overexpression of PGC-1α in skeletal muscle does not alter exercise-induced amelioration of age-dependent decline of hippocampal neurogenesis. **a** Images showing BrdU immunostainings of DG for sedentary and running 11-month-old females 4 weeks after first BrdU injection and start of running, with corresponding graphs (**b**, **c**) representing number of BrdU^+^ cells in the SGZ and GCL for 3- (two-way ANOVA; *n* = 6–10; running effect, *p* < 0.0001) and 11-month-old animals (two-way ANOVA; *n* = 7–9; running effect, *p* = 0.0009). **d** Images of NeuN/BrdU immunostainings of DG for sedentary and running 11-month-old females with confocal images of co-localized NeuN^+^/ BrdU^+^ cells and with corresponding graphs (**e**, **f**) representing number of NeuN^+^/BrdU^+^ cells for 3- (two-way ANOVA; *n* = 5–9; running effect, *p* < 0.0001) and 11-month-old animals (two-way ANOVA; *n* = 7–9; running effect, *p* = 0.05). **g** DCX immunostainings of DG for sedentary and running 11-month-old females with corresponding graphs (**h**, **i**) showing number of DCX^+^ cells for 3- (two-way ANOVA; *n* = 6–10; running effect, *p* < 0.003) and 11-month-old animals (two-way ANOVA; *n* = 5–10; running effect, *p* = 0.002). Data expressed as boxplots using the Tukey method. Scale bars = 20 μm. WT, wild type; Tg, transgenic; Sed, sedentary; Vex, voluntary exercise

We analyzed the number of newly generated BrdU^+^ cells in the subgranular zone (SGZ) and granular cell layer (GCL) of the DG, with respect to genotypes and exercise, using two-way ANOVA for 3- and 11-month-old female animals (see Fig. 3a–c and Table 2). The number of BrdU^+^ cells was decreased with aging and enhanced by exercise in both wild-type and transgenic animals, but no difference existed between genotypes (Tables 2 and 3). The number of newly generated mature neurons determined by NeuN^+^/BrdU^+^ co-labeling was reduced with aging, but exercise did only enhance numbers of new mature neurons in 3-month-old female animals analyzed using two-way ANOVA (see Fig. 3d–f, Tables 2 and 3). Numbers of immature neurons were reduced by aging and enhanced by exercise for both genotypes, but there were no significant differences with respect to genotypes analyzed using two-way ANOVA for 3- and 11-month-old female animals (see Fig. 3g–i, Tables 2 and 3). Additionally, there was no difference in volume of GCL for 3-month-old females (WT sedentary 0.35 ± 0.05 mm^3^, WT runners 0.42 ± 0.08 mm^3^, TG sedentary 0.39 ± 0.08 mm^3^, and TG runners 0.43 ± 0.1 mm^3^; two-way ANOVA, *n* = 6–10; interaction effect, *F*(1,30), *p* = 0.85; genotype effect, *F*(1,30), *p* = 0.32; running effect, *F*(1,30), *p* = 0.12; Online Resource Supplementary Figure S3).

**Table 2:** Genotype, running, and interaction effects of two-way ANOVA on number of newborn cells (BrdU), newborn mature neurons (NeuN/BrdU), and immature neurons (DCX) in the DG

Analysis^a^	Genotype effect	Running effect	Interaction effect
GCL BrdU^+^ cells 3 months^b^	0.26 (*p* = 0.62)	58.1 (*p* < 0.0001)	0.14 (*p* = 0.71)
GCL BrdU^+^ cells 11 months^c^	2.91 (*p* = 0.10)	12.3 (*p* = 0.0016)	0.31 (*p* = 0.58)
Total NeuN^+^/BrdU^+^ 3 months^b^	0.05 (*p* = 0.83)	57.3 (*p* < 0.0001)	0.02 (*p* = 0.91)
Total NeuN^+^/BrdU^+^ 11 months^d^	0.02 (*p* = 0.90)	4.07 (*p* = 0.05)	2.83 (*p* = 0.10)
DCX^+^ cells 3 months^e^	1.55 (*p* = 0.22)	11.1 (*p* = 0.003)	0.83 (*p* = 0.37)
DCX^+^ cells 11 months^f^	0.96 (*p* = 0.34)	11.6 (*p* = 0.002)	0.57 (*p* = 0.46)

^a^See Fig. 3

^b^Data presented as test value of *F*(1, 25) along with the corresponding *p* value in parenthesis (*n* = 5–9)

^c^Data presented as test value of *F*(1, 28) along with the corresponding *p* value in parenthesis (*n* = 7–9)

^d^Data presented as test value of *F*(1, 28) along with the corresponding *p* value in parenthesis (*n* = 7–9)

^e^Data presented as test value of *F*(1, 27) along with the corresponding *p* value in parenthesis (*n* = 6–10)

^f^Data presented as test value of *F*(1, 23) along with the corresponding *p* value in parenthesis (*n* = 5–10)

**Table 3:** Age, genotype, and interaction effects of two-way ANOVA on number of newborn cells (BrdU), newborn mature neurons (NeuN) and immature neurons (DCX) in the DG

Analysis^a^	Age effect	Genotype effect	Interaction effect
GCL BrdU^+^ cells sedentary^b^	260 (*p* < 0.001)	0.40 (*p* = 0.53)	0.002 (*p* = 0.97)
GCL BrdU^+^ cells runners^c^	147 (*p* < 0.001)	0.43 (*p* = 0.52)	0.10 (*p* = 0.75)
Total NeuN^+^/BrdU^+^ sedentary^d^	281 (*p* < 0.001)	0.001 (*p* = 0.97)	0.29 (*p* = 0.60)
Total NeuN^+^/BrdU^+^ runners^e^	147 (*p* < 0.001)	2.22 (*p* = 0.15)	1.68 (*p* = 0.21)
DCX^+^ cells sedentary^f^	63 (*p* < 0.001)	1.30 (*p* = 0.27)	1.50 (*p* = 0.24)
DCX^+^ cells runners^g^	186 (*p* < 0.001)	0.05 (*p* = 0.83)	0.49 (*p* = 0.49)

^a^See Fig. 3

^b^Data presented as test value of *F*(1, 12) along with the corresponding *p* value in parenthesis (*n* = 5–9)

^c^Data presented as test value of *F*(1, 14) along with the corresponding *p* value in parenthesis (*n* = 6–10)

^d^Data presented as test value of *F*(1, 12) along with the corresponding *p* value in parenthesis (*n* = 7)

^e^Data presented as test value of *F*(1, 16) along with the corresponding *p* value in parenthesis (*n* = 9)

^f^Data presented as test value of *F*(1, 12) along with the corresponding *p* value in parenthesis (*n* = 5–7)

^g^Data presented as test value of *F*(1, 13) along with the corresponding *p* value in parenthesis (*n* = 5–10)

**Fig. 4:**
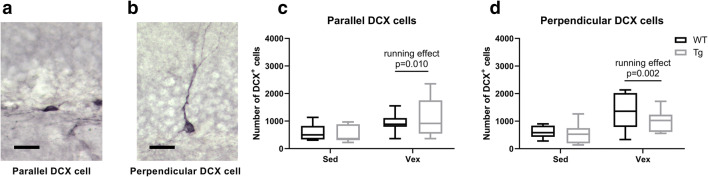
Overexpression of PGC-1α in skeletal muscle does not influence baseline or exercise-induced numbers of **a** immature DCX^+^ cells (oriented in parallel) and **b** mature DCX^+^ cells (perpendicularly oriented) in the DG. Graphs showing number of DCX^+^ cells in DG of sedentary and running 11-month-old females with **c** parallel (two-way ANOVA; *n* = 5–10; running effect, *p* = 0.01) and **d** perpendicular orientation (two-way ANOVA; *n* = 5–10; running effect, *p* = 0.002). Data expressed as boxplots using the Tukey method. Scale bars = 20 μm. WT, wild type; Tg, transgenic; Sed, sedentary; Vex, voluntary exercise

There was a difference in immature DCX^+^ neurons for running versus sedentary female and male, wild-type and transgenic, 11-month-old animals, analyzed using three-way ANOVA (running effect, *F*(1,53) = 11.3, *p* < 0.001), with all other main and interaction effects being non-significant (Online Resource Supplementary Figure S3). However, the number of DCX^+^ cells and running distance was not correlated when analyzing running 11-month-old animals, male and females combined, for either wild-type or transgenic animals (11-month WT runners, *n* = 13, Pearson *r* = 0.47, *p* = 0.11; 11-month TG runners, *n* = 15, Pearson *r* = 0.14, *p* = 0.63; Online Resource Supplementary Figure S3). There were statistically significant differences in numbers of DCX^+^ progenitor with parallel orientation (immature) versus cells with perpendicular orientation (mature) for female 11-month-old animals between sedentary and running, but not between genotypes (see Fig. 4 and Table 4). There was no correlation between number of DCX^+^ cells and running distance for female 11-month-old wild-type or transgenic animals (11-month WT runners: Pearson *r* = 0.69, n.s. [*p* = 0.20], *n* = 5; 11-month TG runners: Pearson *r* = − 0.02, n.s. [*p* = 0.97], *n* = 9).

**Table 4:** Main and interaction effects of two-way ANOVA on number of immature (parallel) and mature (perpendicular) DCX^+^ cells in the DG

Analysis^a^	Genotype effect	Running effect	Interaction effect
Parallel DCX^+^ cells^b^	0.14 (*p* = 0.71)	7.81 (*p* = 0.010)	0.35 (*p* = 0.56)
Perpendicular DCX^+^ cells^b^	1.45 (*p* = 0.24)	11.9 (*p* = 0.002)	1.00 (*p* = 0.33)

^a^See Fig. 4

^b^Data presented as test value of *F*(1, 23) along with the corresponding *p* value in parenthesis (*n* = 5–10)

### Skeletal Muscle PGC-1α Overexpression and Running-Induced Serum Cytokine and Myokine Profile

**Fig. 5:**
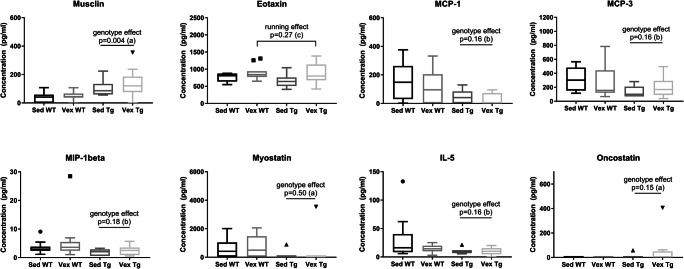
Levels of selected cytokines and myokines in serum from 11-month-old male animals. Graphs showing protein concentrations of analytes with difference or tendency towards differences with genotype and running (see Table 5). Musclin is 4-fold upregulated in transgenic animals. Data expressed as boxplots using the Tukey method. (a) (Scheirer–Ray–Hare extension; *n* = 7–10; FDR-adjusted); (b) (Scheirer–Ray–Hare extension; *n* = 7–11; FDR-adjusted); (***c***) (two-way ANOVA; *n* = 7–11; FDR-adjusted; log transformed). WT, wild type; Tg, transgenic; Sed, sedentary; Vex, voluntary exercise

To investigate possible differences in serum proteins associated with overexpression of PGC-1α in skeletal muscle, running, or a combination of both, we housed 10-month-old male wild-type and transgenic animals individually and subjected them to voluntary running during 4 weeks. After 4 weeks of running, blood was extracted to determine levels of cytokines, chemokines, and myokines in serum. Analysis after FDR adjustment for analyte data shows that the protein musclin is significantly upregulated at 4-fold higher concentration in male transgenic serum (see Fig. 5, Table 5, and Online Resource Supplementary Tables S1, S2, and S3). However, several analytes with significant values before FDR adjustments showed only a trend after FDR and will require further confirmation in future experiments. These include upregulation of eotaxin with running, upregulation of oncostatin in transgenic animals, and downregulation of monocyte chemoattractant protein 1 (MCP-1), MCP-3, interleukin-5 (IL-5), macrophage inflammatory protein 1-beta (MIP-1beta), and myostatin in transgenic animals.

**Table 5:** Genotype, running, and interaction effects of two-way parametric and non-parametric ANOVA on serum concentration of selected cytokines, chemokines, and myokines

Analysis^a^	Genotype effect	Running effect	Interaction effect
Musclin^b^	1630, 14.8 (*p* < 0.001 [*p* = 0.004])	25.9, 0.23 (*p* = 0.63 [*p* = 1.04])	6.30, 0.06 (*p* = 0.81 [*p* = 1.07])
Eotaxin^d,e^	1.69 (*p* = 0.20 [*p* = 0.33])	5.40 (*p* = 0.027 [*p* = 0.27])	0.31 (*p* = 0.58 [*p* = 1.16])
MCP-1^c^	769, 6.73 (*p* = 0.01 [*p* = 0.16])	110, 0.96 (*p* = 0.33 [*p* = 1.54])	0.10, 0.07 (*p* = 0.98 [*p* = 1.04])
MCP-3^c^	623, 5.04 (*p* = 0.02 [*p* = 0.16])	0.20, 0.002 (*p* = 0.97 [*p* = 1.03])	279.2, 2.26 (*p* = 0.13 [*p* = 2.19])
MIP-1beta^c^	716, 5.80 (*p* = 0.02 [*p* = 0.18])	91.7, 0.74 (*p* = 0.39 [*p* = 0.99])	1.50, 0.01 (*p* = 0.91 [*p* = 1.08])
Myostatin^b^	442, 5.66 (*p* = 0.017 [*p* = 0.50])	6.73, 0.09 (*p* = 0.77 [*p* = 1.02])	4.17, 0.05 (*p* = 0.82 [*p* = 1.04])
IL-5^c^	552, 4.47 (*p* = 0.03 [*p* = 0.16])	11.5, 0.09 (*p* = 0.76 [*p* = 1.05])	23.8, 0.19 (*p* = 0.66 [*p* = 1.15])
Oncostatin 5^b^	161, 4.90 (*p* = 0.027 [*p* = 0.15])	16.7, 0.50 (*p* = 0.48 [*p* = 1.13])	19.2, 0.58 (*p* = 0.45 [*p* = 1.05])

^a^See Fig. 5

^b^Scheirer–Ray–Hare extension. Data presented as SS, *H* value along with the corresponding *p* value in parenthesis and FDR-adjusted *p* value in brackets (df = 1, residuals = 32; *n* = 7–10)

^c^Scheirer–Ray–Hare extension. Data presented as SS, *H* value along with the corresponding *p* value in parenthesis and FDR-adjusted *p* value in brackets (df = 1, residuals = 34; *n* = 7–11)

^d^Two-way ANOVA. Data presented as *F*(1,34) with the corresponding *p* value in parenthesis and FDR-adjusted *p* value in brackets (*n* = 7–11)

^e^Log-transformed data

We also examined whether a correlation between running distances and protein levels exists by using linear correlation analysis for normally distributed data and Kendall rank correlation for non-normally distributed data. Similarly, eotaxin shows a positive correlation to running distance, but only in the transgenic group (see Table 6 and Online Resource Supplementary Tables S4 and S5). However, after FDR adjustments, this correlation is no longer significant.

**Table 6:** Linear and Kendall rank correlations of running distance to protein concentration for selected analytes in wild-type and transgenic animals

Analysis^a^	WT			TG		
	Coefficient	*p* value	FDR-adjusted *p* value	Coefficient	*p* value	FDR-adjusted *p* value
Musclin^b,e^	0.15	0.38	0.89	0.06	0.73	0.97
Eotaxin^c,d,f^	0.26	0.50	0.88	0.75	0.01	0.08
MCP-1^b,f^	− 0.08	0.63	0.89	− 0.04	0.85	0.85
MCP-3^b,f^	− 0.18	0.29	1.02	0.32	0.09	0.36
MIP-1beta^b,f^	0.07	0.68	0.79	0.11	0.57	1.14
Myostatin^b,e^	0.02	0.92	0.92	− 0.12	0.59	0.94
IL-5^b,f^	− 0.18	0.29	2.03	0.04	0.83	0.95
Oncostatin 5^b,e^	NA	NA	NA	0.17	0.42	1.12

^a^See Fig. 5

^b^Kendall rank correlation

^c^Linear correlation

^d^Log-transformed data

^e^*n* = 9–10

^f^*n* = 10–11

Finally, the relationship between number of DCX^+^ cells and eotaxin has previously been reported as being negatively correlated in middle-aged animals [42, 43], while we find a positive correlation with eotaxin serum levels in 11-month-old male transgenic, but not in wild-type animals (linear correlation; WT: *r* = 0.06, n.s. [*p* = 0.85], *n* = 11; TG: *r* = 0.68, *p* = 0.005, *n* = 15).

## Discussion

In this study, we have investigated the influence of muscular overexpression of PGC-1α on exercise-induced neurogenesis. We find that muscular overexpression of PGC-1α does not influence exercise-induced neurogenesis, irrespective of age of the animal, indicating that peripheral PGC-1α overexpression is not sufficient to induce neurogenesis nor to enhance exercise-induced effects on neurogenesis.

The transgenic mouse model used in this study has a moderate overexpression of PGC-1α and its downstream effector genes *Fndc5*, *Vegfb*, *IL15*, *Timp4*, and *Ctsb* [34, 35] and displays an endurance muscle phenotype with high mitochondrial density [34]. In this study, we observed a 3–4-fold increase in PGC-1α protein expression in gastrocnemius of transgenic sedentary female and male animals. After 4 weeks of voluntary running, the difference in gastrocnemius PGC-1α protein expression between wild-type and transgenic female animals was no longer detectable, suggesting a higher relative increase in gastrocnemius PGC-1α protein levels from endurance exercise in wild-type animals compared to in MCK-PGC-1α animals. This could be related to the muscle creatinine kinase promoter element in the transgenic construct not being further regulated by exercise. However, we have not been able to statistically confirm an increase in gastrocnemius PGC-1α protein levels from exercise in female animals, due to differences in sample preparation between sedentary and exercised animals (as can be seen by differences in total protein staining), or in male animals, due to insufficient running activity.

### Muscle-Specific Overexpression of PGC-1α Does Not Impact Voluntary Running Activity

Running distance observed in this study is comparable to previous reports on running distance in C57BL/6J mice [44]. We observed a big difference between running in young and old animals, as well as a smaller difference between females and males. Reduced running activity in aging is related to a decrease in running velocity, with unchanged duration of running intervals compared to younger animals [45]. This reduction in running velocity could represent a decrease in muscle strength due to reduced muscle mass and muscle fibrosis with aging [46].

For C57BL6 mice, as for other strains, females are known to run for longer distances than males due to higher velocities, but for the same duration as males [47], possibly due to better respiratory capabilities in female mice [48] and differences in energy metabolism [49]. Bartling and colleagues reported that the difference in running activity for females and males gradually diminish with age [47], which was attributed to age-related changes in metabolism [49] and hormonal regulation [50].

Aging reduces exercise-induced adaptations in the muscle, with diminished induction of PGC-1α, nuclear respiratory factor-1 (NRF-1), and cytochrome c [51] to levels comparable to that of PGC-1α knock-out mice. These age-related changes in muscle can be ameliorated by overexpression of PGC-1α in muscle, which rejuvenates aging tissue and enhances a subset of molecular patterns similar to young animals [52]. However, we did not observe any difference in voluntary running activity between genotypes, which stands in contrast to the enhanced endurance capacity that these transgenic animals have been reported to display. Calvo and colleagues reported that overexpression of PGC-1α in muscle greatly improved short-term exercise performance in a voluntary exercise paradigm [53] when measured by single housing animals with running wheels for 72 h. In our data, we see that voluntary running activity increases substantially over 4 weeks, a period over which no differences were detected between genotypes. However, MCK-PGC-1α mice has been reported to outperform wild-type animals in a forced exercise paradigm due to a higher peak oxygen uptake in muscle [53].

### Muscle-Specific Overexpression of PGC-1α Does Not Contribute to Exercise-Induced Neurogenesis

It is well-known that neurogenesis is reduced with aging [54, 55]. Voluntary exercise is a potent enhancer of hippocampal neurogenesis and upregulates several neurotrophic growth factors in the hippocampus [29, 56, 57]. The neurogenic response to running results in a 2–3-fold increase in new neurons, depending on genetic background [58, 59], age [5], running wheel type [60], and running distance [61]. With exercise, we observed a robust increase in neurogenesis in terms of newborn BrdU^+^ cells, new immature DCX^+^ neurons, and mainly for 3-month-old animals, new mature BrdU^+^/NeuN^+^ neurons. There is a trend towards female having more DCX cells, possibly due to the fact that they engage more intensely in voluntary wheel running. A limitation of the study is that we only have BrdU and BrdU/NeuN data from female animals. Therefore, extending the interpretation of the data to males should be done with caution, especially since females and males show different running wheel activity.

Endurance exercise leads to changes in maturation, morphology, and connectivity, shortening the cell cycle of rapidly proliferation progenitor cells [62] and accelerating maturation of adult-born DG neurons [63]. The orientation of DCX^+^ cells in the DG can be used for differentiating between a more immature versus a more mature progenitor state [64]. We did not detect any difference in orientation of DCX^+^ cells between genotypes, indicating no impact of muscular PGC-1α overexpression on neuronal maturation in hippocampal neurogenesis.

Based on the previously reported upregulation of systemic factors with neurotrophic effects in MCK-PGC-1α mice [12, 17], we hypothesized that constitutive muscular PGC-1α overexpression could result in increased baseline neurogenesis and that endurance exercise may further add to these effects. In our study, we were unable to detect any additional effect of transgenic overexpression of PGC-1α in muscle beyond the physiological levels of baseline or exercise-induced neurogenesis in young or old mice. This is in alignment with a study showing no further improvement of metabolic enhancements from exercise by muscle-specific PGC-1α overexpression [65]. MCK-PGC-1α mice have also been reported to be even more prone to fat-induced insulin resistance due to decreased insulin sensitivity in muscle [66] rather than providing protection against insulin resistance.

### Muscle-Specific Overexpression of PGC-1α Increases Serum Levels of Musclin

We measured cytokines, chemokines, and myokines in serum of 11-month-old male animals, using commercially available multiplex immunoassay kits. We prioritized analyzing older animals since we assumed we would see the biggest influence of PGC-1α overexpression at this age due to the reduced muscular expression of PGC-1α with aging [67]. We collected and analyzed serum during the inactive phase of the animals to reduce the influence of acute exercise on induction of circulating proteins. It is known that acute and chronic exercise differentially regulate proteins in the circulation [68]. Measurements in the inactive phase could be viewed as reflecting long-term tissue adaptations due to repeated regular exercise instead of the stress-like response due to an acute exercise bout. Our hypothesis was that chronic changes would better explain the genotypes differences.

We observed the myokine musclin (also known as the bone-derived peptide osteocrin) to be upregulated to 4-fold higher concentrations compared to wild type. Garcia and colleagues noted a small increase in musclin and fibroblast growth factor 21 (FGF21) in sera from extremely old MCK-PGC-1α mice [52]. Musclin is induced in muscle as a response to exercise and is essential for muscular adaptations to endurance training [69] due to induction of mitochondrial biogenesis in muscle [70]. Musclin shares structural homology with cardiac natriuretic peptide that acts on blood vessel and kidneys and is involved in mitochondrial biogenesis, angiogenesis, lipolysis, and adipose tissue remodeling [69], suggesting a possible systemic function on other tissues as well. Musclin has been implicated in human cognition through inhibition of dendritic growth in an activity-dependent manner by binding to myocyte enhancer factor 2 (MEF2) [71]. Whether peripheral or endogenous brain expression of musclin is primarily responsible for its CNS effect is however an open question. We found that serum levels of pro-inflammatory mediators MCP-1, MCP-3, IL-5, and MIP-1beta, as well as the myokine myostatin, showed a trend towards downregulation, while oncostatin was upregulated in transgenic animals. However, these differences were not significant after FDR adjustment.

A recent study by Peng and colleagues found that MCK-PGC-1α mice had 2-fold upregulated serum levels of the myokines irisin, BDNF, and IL-15 [28], which are linked to neuroprotection. The commercial multiplex assays employed in our study included quantification of irisin and BDNF in serum. However, the assay was not sensitive enough to enable statistical comparisons between the groups.

We did not observe any significant effect of exercise on the studied serum proteins. This is in accordance with a previous study by Jeon and colleagues who were unable to detect serum differences in a panel of 50 cytokines in either 2- or 20-month-old mice after 4 weeks of treadmill running [72]. However, they observed an age-dependent upregulation of eotaxin, IL-9 and CCL17 when comparing 2-month-old to 20-month-old mice. We detected a trend towards upregulation of eotaxin in serum with running, an exercise-induced chemokine that has the ability to cross the blood–brain barrier [73, 74]. However, this difference was not significant after adjustment for FDR. Eotaxin has also been identified as one of the factors inhibiting neurogenesis during aging [42]. A previous study found that the number of hippocampal DCX cells was negatively correlated with plasma eotaxin levels in 21-month-old mice [75]. In our study, we instead found a positive correlation between eotaxin and number of DCX^+^ cells for transgenic running animals, supporting an alternate role for eotaxin as a possible exercise-induced neuromodulator.

Leiter and colleagues, found that 38 proteins were upregulated in mouse plasma after 4 days of voluntary running using tandem mass spectrometry [76]. In another study by Little and colleagues, the authors analyzed 66 cytokines by multiplex and did not find any upregulated proteins in serum after 1 week of voluntary running. Instead they found reduced serum concentrations of TIMP-1, MIP-3alpha, IL-16, IFN-gamma, and G-CSF [68]. Interestingly, platelets are transiently activated after acute periods of running, with the release of platelet-associated proteins into plasma and promotion of hippocampal neurogenesis [76]. Preparation of serum involves a clotting process in which platelets are activated and release their content. This release of platelet content may conceal changes in circulating proteins by exercise-induced activation of platelets. Therefore, it is likely that the proteomics profiling after long-term exercise employed in this study would have yielded bigger differences with mouse plasma instead of serum as substrate for multiplex analysis.

In a study by De Miguel and colleagues, plasma from voluntary running 3-month-old mice injected in age-matched sedentary animals resulted in increased hippocampal neurogenesis and improvement in memory and learning [77]. This effect was most pronounced with plasma from 28-day-runners which increased cell proliferation, survival and neuroblast count, whereas plasma from 7- and 14-day-runners only resulted in an increased neuroblast count. The authors identified 23 downregulated and 26 upregulated proteins in plasma from running animals using tandem mass spectrometry, overall indicating an effect on the complement and coagulation cascades in the blood. Further, they found that plasma from running animals could reverse neuroinflammatory response to LPS in the hippocampus, an effect dependent on clusterin, an inhibitor of the complement system.

A limitation of the study is that we, due to technical considerations, decided against performing the morphological analysis and serum protein analysis in the same sex, limiting direct comparison of neurogenesis and serum responses to exercise. Further, based on the number of analytes chosen in the multiplex analysis (i.e., 34), a significance level of 0.05 before correction for repeated significance test, and a sample size of 36 animals, our post hoc calculation indicates that we have an approximate probability of 30% to detect an effect size of the same magnitude as muslin (i.e., effect size of 0.48). Due to the multiplex analysis being underpowered, we cannot rule out that there are other important differences influenced by genotype or running in the analytes measured other than an upregulation of serum musclin in male transgenic animals. For this reason, the serum protein analysis data should mainly be considered as exploratory, i.e., hypothesis generating, rather than hypothesis confirming.

### Mimicking Exercise-Induced Effects on the CNS

Exercise induces PGC-1α expression in both skeletal muscle and brain [17, 78]. One of the prominent effects of elevated PGC-1α is the increased expression of FNDC5, which is cleaved and released as irisin into the circulation [12]. An acute peripheral overexpression of FNDC5 has been reported to increased BDNF expression in the hippocampus [17]. Recently, peripheral overexpression of FNDC5/irisin by intravenous application of an adenoviral vector was reported to impact memory impairment and improve synaptic plasticity in a mouse model of Alzheimer’s disease [18]. However, the lack of effect we see in the CNS of MCK-PGC-1α animals is highly relevant in the prediction of sustainable effects for other indications concerning the activation of the PGC1/FNDC5 pathway. Our data show that upregulation of the PGC1/FNDC5 pathway in muscle is not sufficient to achieve exercise-induced effects on the CNS and that upregulation in other tissues or other effects might be necessary for this purpose [17, 18].

Other pharmacological treatments of muscle activation have shown limited ability to mimic exercise-induced neurogenesis. The substance 5-aminoimidazole-4-carboxamide ribonucleotide (AICAR) is a pharmacological mimetic that can upregulate the AMPK-pathway and can lead to a temporary boost in neurogenesis [79]. Combined pharmacological increase of adult neurogenesis by AICAR and BDNF was able to mimic exercise-induced effects on cognition in an Alzheimer’s mouse model [80]. Both studies, by Choi and by Lourenco, have observed short-term effects of treatment up to a 2-week time point. In contrast to these acute models of supraphysiological pathway stimulation, our chronic muscle activation model provides an estimate of expected long-term effects from molecular targeting of exercise-induced pathways, factoring in compensatory counter-regulation that may occur over time.

## Conclusion

We conclude that forced expression of the PGC-1α pathway in skeletal muscle is, despite potent systemic changes, not sufficient to mimic exercise-induced neurogenesis. Further studies are required to determine additional signals from muscle and other tissue that could be important contributors to exercise-inducing changes in the brain. Exercise induces complex effects through a range of different pathways, and sustainable exercise-mimicking effects on neuroprotection and neuroplasticity most likely will need to target more than one pathway.

## Supplementary Information

Figure S1 (PNG 1080 kb)

High Resolution (TIFF 10355 kb)

Figure S2 (PNG 849 kb)

High Resolution (TIFF 8206 kb)

Figure S3 (PNG 66 kb)

High Resolution (TIF 1457 kb)

Figure S4 (PNG 146 kb)

High Resolution (TIF 1218 kb)

ESM 1 (DOCX 42 kb)

## Abbreviations

AICAR: 5-Aminoimidazole-4-carboxamide ribonucleotide
AMPK: Adenosine monophosphate-activated protein kinase
ANOVA: Analysis of variance
BDNF: Brain-derived neurotrophic factor
BrdU: Bromodeoxyuridine
CCL17: Chemokine (C-C motif) ligand 17
CNS: Central nervous system
CTSB: Cathepsin B
DCX: Doublecortin
DG: Dentate gyrus
FDR: False discovery rate
FGF21: Fibroblast growth factor 21
FNDC5: Fibronectin type III domain-containing protein 5
GCL: Granular cell layer
IGF-1: Insulin-like growth factor-1
IL-5: Interleukin-5
MAPK: Mitogen-activated protein kinase
MCK-PGC-1α: Transgenic mice overexpressing PGC-1α under muscle creatinine kinase promoter
MCP-1/3: Monocyte chemoattractant protein-1/3
MEF2: Myocyte enhancer factor 2
MIP-1beta: Macrophage inflammatory protein-1beta
ML: Molecular layer
NeuN: Neuronal nuclei
NRF-1: Nuclear respiratory factor-1
PGC-1α: Peroxisome proliferator-activated receptor gamma co-activator 1-alpha
RT: Room temperature
SGZ: Subgranular zone
TG: Transgenic
TIMP4: Metalloproteinase inhibitor 4
VEGF: Vascular endothelial growth factor
WT: Wild type
