# Supplementary figures and images for: Constitutive PGC-1α Overexpression in Skeletal Muscle Does Not Contribute to Exercise-Induced Neurogenesis
Source: Mol Neurobiol. 2020 Nov 16;58(4):1465–81. doi: 10.1007/s12035-020-02189-6 (PMC7932943; doi:10.1007/s12035-020-02189-6)

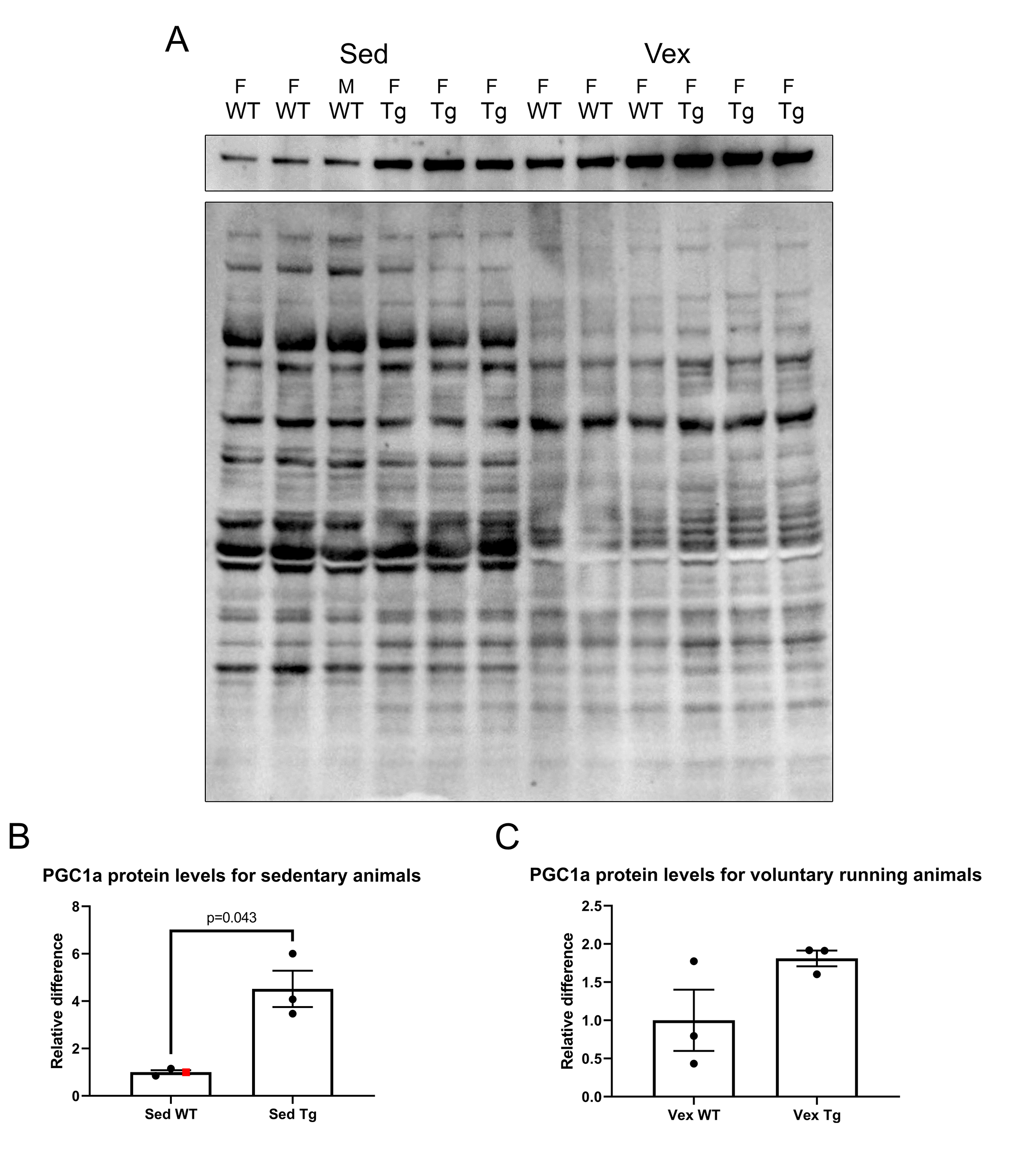

Supplement: Supplementary file 1 — (PNG 1080 kb) [file 12035_2020_2189_Fig6_ESM.png]

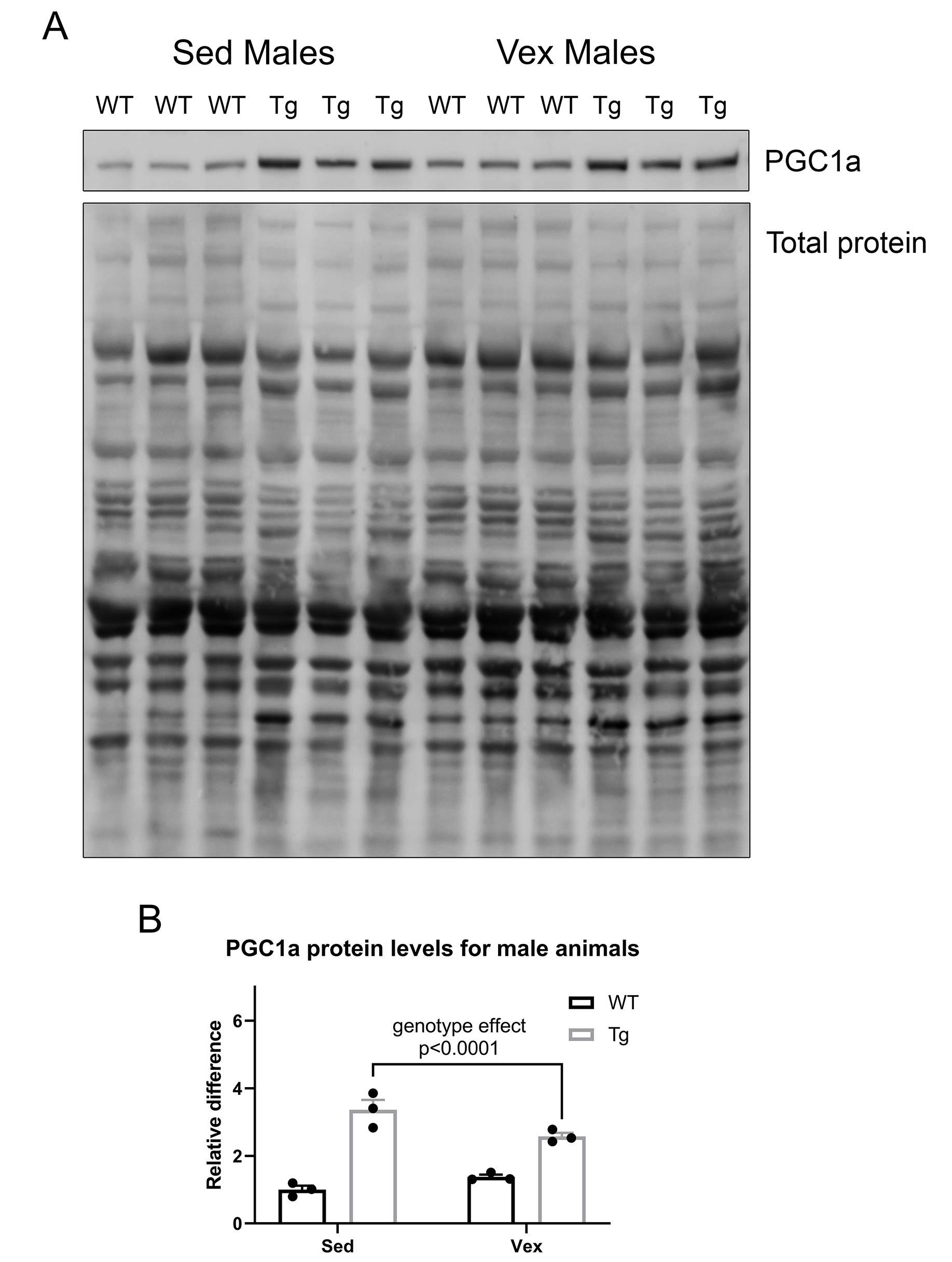

Supplement: Supplementary file 3 — (PNG 849 kb) [file 12035_2020_2189_Fig7_ESM.png]

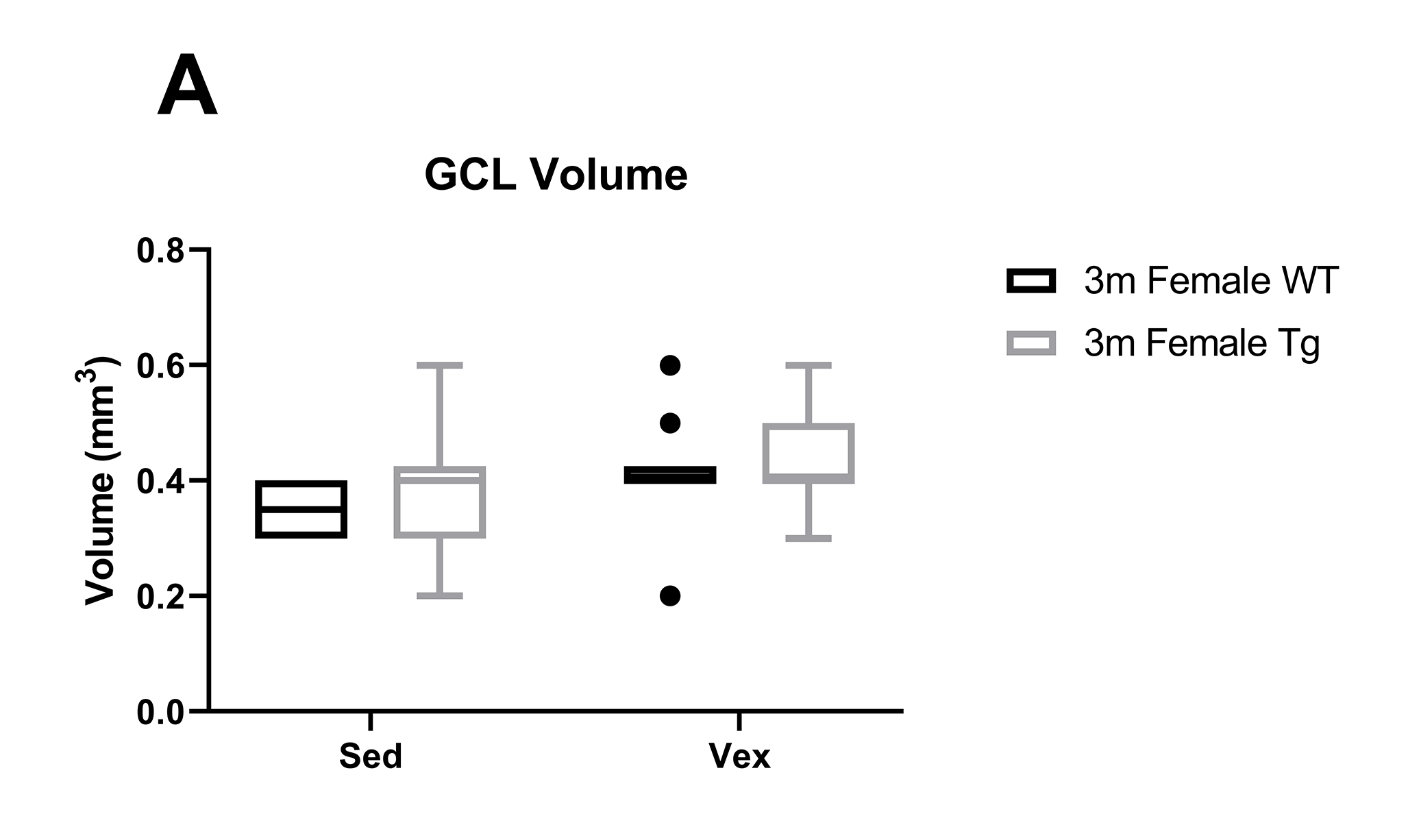

Supplement: Supplementary file 5 — (PNG 66 kb) [file 12035_2020_2189_Fig8_ESM.png]

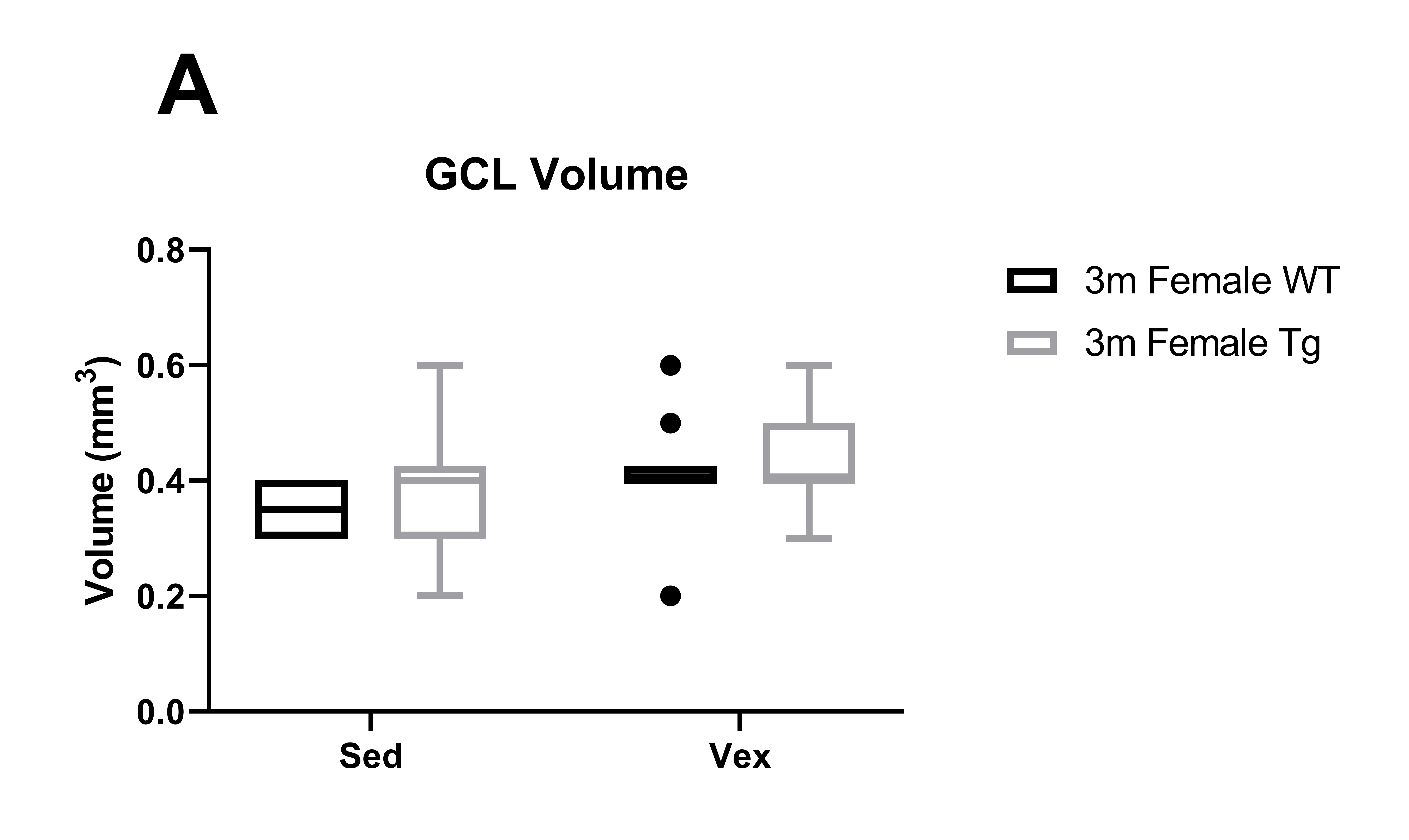

Supplement: Supplementary file 6 — High Resolution (TIF 1457 kb) [file 12035_2020_2189_MOESM3_ESM.tif]

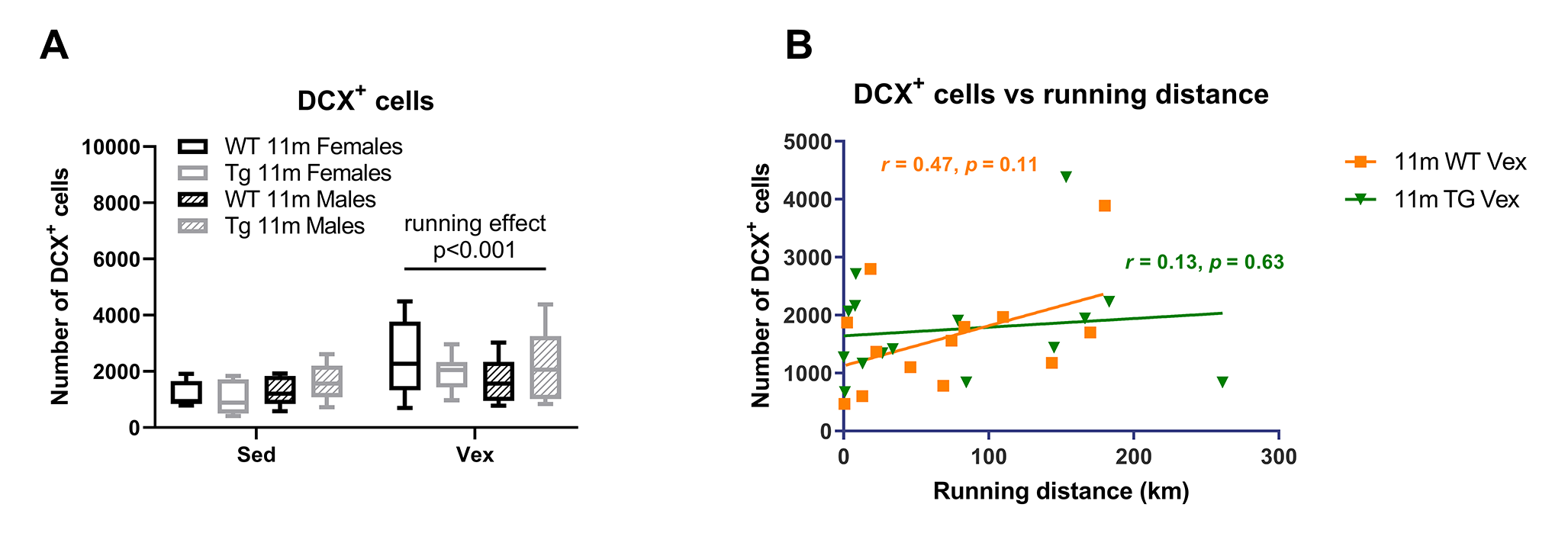

Supplement: Supplementary file 7 — (PNG 146 kb) [file 12035_2020_2189_Fig9_ESM.png]

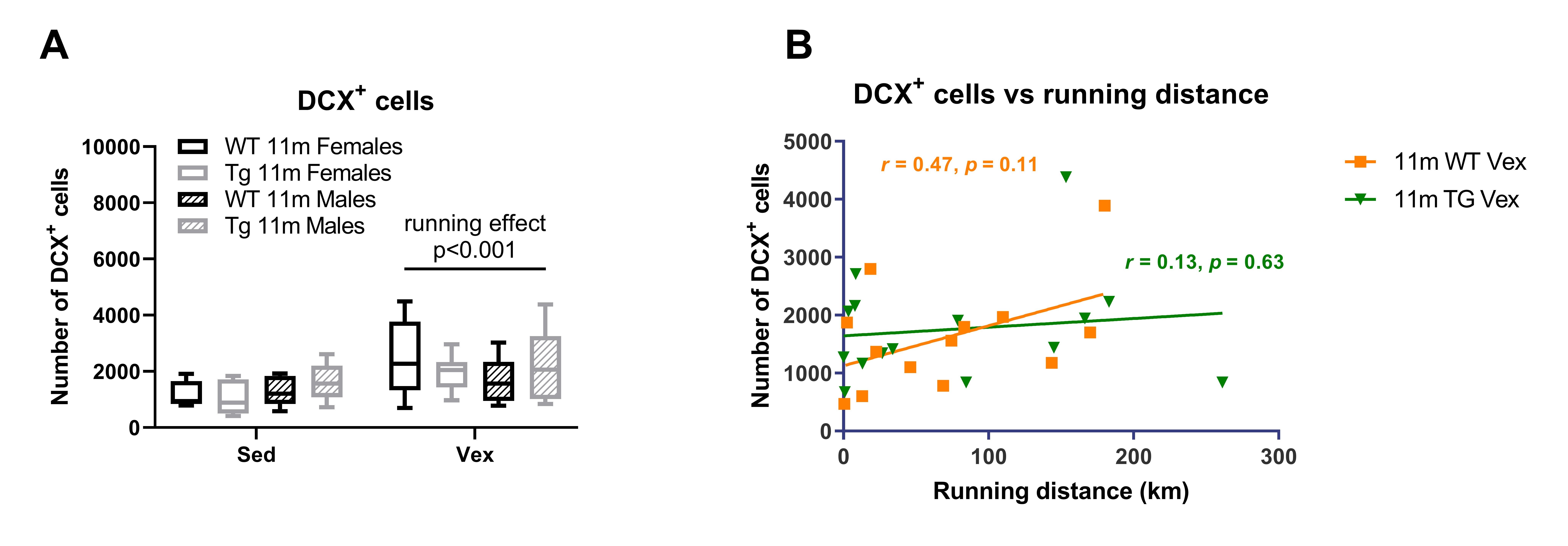

Supplement: Supplementary file 8 — High Resolution (TIF 1218 kb) [file 12035_2020_2189_MOESM4_ESM.tif]
