# Supplementary material for: Constitutive PGC-1α Overexpression in Skeletal Muscle Does Not Contribute to Exercise-Induced Neurogenesis
Source: Mol Neurobiol. 2020 Nov 16;58(4):1465–81. doi: 10.1007/s12035-020-02189-6 (PMC7932943; doi:10.1007/s12035-020-02189-6)
Supplement: Supplementary file 9 — (DOCX 42 kb) [file 12035_2020_2189_MOESM5_ESM.docx]

# Constitutive PGC-1α Overexpression in Skeletal Muscle Does Not Contribute to Exercise-Induced Neurogenesis

Lars Karlsson*^1,2^, María Nazareth González-Alvarado^1^, Yafeng Wang^1,3,4^, Reza Motalleb^1^, Changlian Zhu^1,3^, Mats Börjesson^5,6^, Hans-Georg Kuhn^1^

^1^Institute for Neuroscience and Physiology, University of Gothenburg, Gothenburg, Sweden.

^2^The Queen Silvia Children’s Hospital, Sahlgrenska University Hospital, Gothenburg, Sweden.

^3^Henan Key Laboratory of Child Brain Injury, Institute of Neuroscience and Third Affiliated Hospital of Zhengzhou University, Zhengzhou, China

^4^Department of Pediatrics, Children’s Hospital Affiliated to Zhengzhou University, Zhengzhou, China

^5^Dept of Molecular and Clinical Medicine, Sahlgrenska Academy and Center for Health and Performance, University of Gothenburg, Gothenburg, Sweden.

^6^Sahlgrenska University Hospital/Östra, Gothenburg, Sweden.

*Correspondence:

E-mail: [lars.karlsson@neuro.gu.se](mailto:lars.karlsson@neuro.gu.se)

Postal address: Center for Brain Repair and Rehabilitation, Institute for Neuroscience and Physiology, University of Gothenburg, Medicinargatan 11 (S-floor), 413 90 Gothenburg, Sweden.

# Supplementary Figure Legends

**Supplementary Figure 1.** Overexpression of PGC-1α in skeletal muscle leads to increased PGC-1α protein levels in skeletal muscle. Images shows western blot on gastrocnemius homogenate from wildtype and transgenic MCK- PGC-1α animals for (**a**) 7-10-month-old sedentary animals and after 4 weeks of voluntary running. Graphs shows PGC1a protein levels normalized to total protein for (**b**) sedentary animals (red dot indicates a male animal as shown in a; t-test with Welch correction, n=3, p=0.043) and (**c**) voluntary running animals (t-test with Welch correction, n=3, n.s. [p=0.174]). Normalized PGC-1α protein levels are shown relative to the mean of the corresponding sedentary or running wildtype group. Sedentary and wildtype groups have not been compared against each other due to differences in total protein staining between the samples. Data expressed as mean ± SEM.

**Supplementary Figure 2.** Overexpression of PGC-1α in skeletal muscle leads to increased PGC-1α protein levels in skeletal muscle. Images shows western blot on gastrocnemius homogenate from 7-month-old male wildtype and transgenic MCK-PGC-1α animals for (**a**) under a sedentary condition and after 4 weeks of voluntary running. Graph shows PGC-1α protein levels normalized to total protein for (**b**) sedentary and running animals. Normalized PGC-1α protein levels are shown relative to the mean of the sedentary wildtype group. Two-way ANOVA shows a genotype effect, p<0.0001 (n=3), with running and interaction effects non-significant. Data expressed as mean ± SEM.

**Supplementary Figure 3.** Overexpression of PGC-1α in skeletal muscle does not influence DG volume. Graph showing volume of the GCL (two-way ANOVA, n=6-10, n.s.) in 3-month-old female animals after 4 weeks of voluntary running. Data expressed as boxplots using the Tukey method.

**Supplementary Figure 4.** Overexpression of PGC-1α in skeletal muscle does not influence number of DCX^+^ cells after 4 weeks of voluntary running. Graph showing (**a**) number of DCX^+^ cells for sedentary and running, female and male, wildtype and transgenic 11-month-old animals (3-way ANOVA; n=5-10), and (**b**) no correlation exists between the number of DCX^+^ cells and running distance for 11-month-old animals (linear correlation; n=14-17; n.s.). Data expressed as boxplots using the Tukey method.

# Supplementary Tables

**Supplementary Table 1.** Proteomic profiling of serum concentration of cytokines, chemokines, and myokines, in transgenic and wildtype animals and in response to 4 weeks of voluntary running. Data expressed in unit of concentration pg/mL as mean ± SEM. *WT, wildtype; TG, transgenic;* *Sed, sedentary; Vex, voluntary exercise.*

| **Analyte** | **WT sed** | **WT vex** | **TG sed** | **TG vex** |
| --- | --- | --- | --- | --- |
| Brain-derived neurotrophic factor (BDNF) | 3.20 ± 1.58^a^ | 1.77 ± 1.3^b^ | 0.9 ± 0.5^d^ | 0.52 ± 0.3^a^ |
| Fibroblast growth factor 21 (FGF21) | 196.8 ± 33.1^a^ | 115.7 ± 44.2^b^ | 144.3 ± 28.3^d^ | 136.9 ± 37.7^a^ |
| Fractalkine (CX3CL1) | 9 ± 0^a^ | 9 ± 0^b^ | 9 ± 0^d^ | 9 ± 0^a^ |
| Follistatin-like 1 (FSTL1) | 50 ± 0^a^ | 83.6 ± 31.64^b^ | 50 ± 0^d^ | 50 ± 0^a^ |
| Myostatin | 605.4 ± 204.1^a^ | 732.6 ± 250.2^b^ | 162.2 ± 113.1^d^ | 390.9 ± 332.8^a^ |
| Irisin | 33.7 ± 28.2^a^ | 4 ± 0^b^ | 4 ± 0^d^ | 114.2 ± 74.3^a^ |
| Oncostatin 5 | 1.1 ± 0^a^ | 1.1 ± 0^b^ | 9.15 ± 7.45^d^ | 52.2 ± 37.9^a^ |
| Osteocrin (Musclin) | 37 ± 11.1^a^ | 50.5 ± 9.12^b^ | 109 ± 20.4^d^ | 138.3 ± 29.9^a^ |
| Osteonectin | 26,219 ± 4,546^a^ | 23,177 ± 3,746^b^ | 22,225 ± 2,539^d^ | 24,879 ± 2,083^a^ |
| ENA-78 (CXCL5) | 765.6 ± 120.1^a^ | 963.8 ± 102.1^c^ | 594.9 ± 69.7^d^ | 798.8 ± 152.0^a^ |
| Eotaxin (CCL11) | 766.2 ± 37.3^a^ | 900.06 ± 59.5^c^ | 667.9 ± 70.2^d^ | 871.7 ± 89.6^a^ |
| Granulocyte colony-stimulating factor (G-CSF) | 11.5 ± 3.28^a^ | 9.44 ± 1.63^c^ | 9.5 ± 1.13^d^ | 6.81 ± 1.38^a^ |
| Granulocyte-macrophage colony-stimulating factor  (GM-CSF) | 1.5 ± 0.74^a^ | 3.19 ± 2.95^c^ | 0.1 ± 0^d^ | 0.59 ± 0.46^a^ |
| Growth-regulated oncogene-alpha (GRO-alpha/CXCL1) | 115 ± 43.4^a^ | 42.7 ± 7.64^c^ | 30.7 ± 8.42^d^ | 34.9 ± 9.7^a^ |
| Interferon-alpha (IFN-alpha) | 1.52 ± 0^a^ | 1.52 ± 0^c^ | 1.52 ± 0^d^ | 1.52 ± 0^a^ |
| Interferon-gamma (IFN-gamma) | 1.2 ± 0.39^a^ | 0.8 ± 0.39^c^ | 0.71 ± 0.29^d^ | 0.94 ± 0.42^a^ |
| Interleukin 1alpha (IL-1alpha) | 9.4 ± 2.45^a^ | 9.67 ± 2.27^c^ | 5.8 ± 1.85^d^ | 5.93 ± 1.89^a^ |
| Interleukin 1beta (IL-1beta) | 1.2 ± 0.49^a^ | 1.55 ± 0.89^c^ | 0.34 ± 0.25^d^ | 0.51 ± 0.28^a^ |
| Interleukin 10 (IL-10) | 23 ± 6.20^a^ | 19.3 ± 4.9^c^ | 12.4 ± 2.38^d^ | 11.6 ± 4.77^a^ |
| Interleukin 12p70 (IL-12p70) | 0.1 ± 0^a^ | 0.41 ± 0.28^c^ | 0.11 ± 0^d^ | 0.11 ± 0^a^ |
| Interleukin 13 (IL-13) | 8.6 ± 7.59^a^ | 1.47 ± 1.33^c^ | 0.08 ± 0^d^ | 2.13 ± 1.55^a^ |
| Interleukin 15 (IL-15) | 1.1 ± 0.46^a^ | 1.16 ± 0.74^c^ | 0.21 ± 0^d^ | 0.96 ± 0.72^a^ |
| Interleukin 17 (IL-17) | 1.6 ± 0.81^a^ | 1.89 ± 1.62^c^ | 1.37 ± 0.67^d^ | 1.98 ± 1.32^a^ |
| Interleukin 18 (IL-18) | 242.4 ± 49.3^a^ | 436.1 ± 110.1^c^ | 185.1 ± 25.3^d^ | 212.5 ± 38.6^a^ |
| Interleukin 2 (IL-2) | 0.2 ± 0.17^a^ | 1.63 ± 1.51^c^ | 0.05 ± 0^d^ | 0.05 ± 0^a^ |
| Interleukin 22 (IL-22) | 76 ± 26.9^a^ | 62.1 ± 16.9^c^ | 39.3 ± 7.03^d^ | 44.4 ± 11.9^a^ |
| Interleukin 23 (IL-23) | 102.1 ± 26.6^a^ | 74.4 ± 11.1^c^ | 79.2 ± 12.76^d^ | 63.5 ± 11.2^a^ |
| Interleukin 27 (IL-27) | 12.8 ± 3.01^a^ | 8.81 ± 1.99^c^ | 8.79 ± 1.98^d^ | 7.4 ± 2.28^a^ |
| Interleukin 28 (IL-28) | 29.9 ± 8.21^a^ | 57.6 ± 29.5^c^ | 45.2 ± 19.3^d^ | 22.8 ± 6.37^a^ |
| Interleukin 3 (IL-3) | 0.1 ± 0^a^ | 0.12 ± 0.06^c^ | 0.06 ± 0^d^ | 0.1 ± 0.04^a^ |
| Interleukin 31 (IL-31) | 0.2 ± 0^a^ | 6.57 ± 6.04^c^ | 0.23 ± 0^d^ | 0.23 ± 0^a^ |
| Interleukin 4 (IL-4) | 3.4 ± 0.58^a^ | 3.83 ± 0.45^c^ | 1.78 ± 0.64^d^ | 3.44 ± 0.72^a^ |
| Interleukin 5 (IL-5) | 31.7 ± 11.8^a^ | 14.3 ± 1.93^c^ | 10.5 ± 1.86^d^ | 10.1 ± 2.05^a^ |
| Interleukin 6 (IL-6) | 80.2 ± 39.3^a^ | 55.9 ± 33.0^c^ | 10.0 ± 9.18^d^ | 32.4 ± 24.6^a^ |
| Interleukin 9 (IL-9) | 44.3 ± 22.7^a^ | 43.7 ± 13.8^c^ | 22.1 ± 13.8^d^ | 34.3 ± 22.7^a^ |
| Interferon gamma-induced protein 10 (IP-10/CXCL10) | 52 ± 6.88^a^ | 62.9 ± 6.51^c^ | 41.7 ± 5.29^d^ | 50.0 ± 6.34^a^ |
| Leukemia inhibitory factor (LIF) | 6.1 ± 1.01^a^ | 8.55 ± 1.26^c^ | 8.39 ± 1.03^d^ | 7.56 ± 1.06^a^ |
| Monocyte chemoattractant protein-1 (MCP-1/CCL2) | 148.8 ± 38.4^a^ | 112.0 ± 34.2^c^ | 46.7 ± 17.4^d^ | 26.2 ± 12^a^ |
| Monocyte chemoattractant protein-3 (MCP-3/CCL7) | 315.1 ± 51.2^a^ | 276.4 ± 64.3^c^ | 130.9 ± 28.7^d^ | 199.8 ± 41.3^a^ |
| Macrophage colony-stimulating factor (M-CSF) | 0 ± 0^a^ | 0.1 ± 0.09^c^ | 0.01 ± 0^d^ | 0.01 ± 0^a^ |
| Macrophage inhibitory protein 1alpha (MIP-1alpha/CCL3) | 1.9 ± 1.6^a^ | 3.14 ± 2.41^c^ | 0.07 ± 0^d^ | 0.93 ± 0.74^a^ |
| Macrophage inhibitory protein 1beta (MIP-1beta/CCL4) | 3.6 ± 0.67^a^ | 5.76 ± 2.21^c^ | 1.96 ± 0.38^d^ | 2.41 ± 0.5^a^ |
| Macrophage inhibitory protein 2 (MIP-2/CXCL2) | 22 ± 1.72^a^ | 24.5 ± 1.27^c^ | 22.4 ± 1.75^d^ | 23.3 ± 1.55^a^ |
| RANTES (CCL5) | 48.3 ± 11.7^a^ | 62.0 ± 9.92^c^ | 30.3 ± 4.25^d^ | 45.0 ± 7.57^a^ |
| Tumor necrosis factor alpha (TNF-alpha) | 0.2 ± 0^a^ | 3 ± 2.12^c^ | 0.2 ± 0^d^ | 0.2 ± 0^a^ |

^a^ n=10

^b^ n=9

^c^ n=11

^d^ n=7

**Supplementary Table 2.** Main and interaction effects of two-way parametric ANOVA on serum concentration of cytokines, chemokines, and myokines.

| Analysis  Osteonectin^b^  ENA-78^a,c^  Eotaxin^a,c^  G-CSF^a,c^  GRO-alpha^a,c^  IL-23^a,c^  IL-4^c^  IP-10^a,c^  MIP-2^c^  RANTES^a,c^ | Genotype effect  0.09 (p=0.76 [p=0.84])  2.5 (p=0.12 [p=0.24])  1.7 (p=0.20 [p=0.33])  0.22 (p=0.64 [p=0.91])  3.0 (p=0.09 [p=0.30])  0.11 (p=0.74 [p=0.93])  2.5 (p=0.12 [p=0.30])  3.3 (p=0.08 [p=0.40])  0.06 (p=0.80 [p=0.80])  3.0 (p=0.06 [p=0.60]) | Running effect  0.003 (p=0.96 [p=0.96])  2.5 (p=0.13 [p=0.33])  5.4 (p=0.03 [p=0.27])  0.74 (p=0.40 [p=0.50])  0.31 (p=0.58 [p=0.64])  0.87 (p=0.36 [p=0.51])  2.6 (p=0.12 [p=0.60])  2.1 (p=0.16 [p=0.32])  1.1 (p=0.31 [p=0.52])  2.0 (p=0.12 [p=0.40]) | Interaction effect  0.58 (p=0.45 [p=1.50])  0.11 (p=0.75 [p=1.07])  0.31 (p=0.58 [p=1.16])  1.2 (p=0.28 [p=2.80])  0.33 (p=0.57 [p=1.42])  0.08 (p=0.78 [p=0.87])  0.95 (p=0.34 [p=1.70])  0.10 (p=0.76 [p=0.95])  0.21 (p=0.65 [p=1.08])  0.002 (p=0.94 [p=0.94]) |
| --- | --- | --- | --- |

^a^ Log transformed data.

^b^ Data presented as F(1,32) with the corresponding p-value in parenthesis and FDR-adjusted p-value in brackets (n=7-10).

^c^ Data presented as F(1,34) with the corresponding p-value in parenthesis and FDR-adjusted p-value in brackets (n=7-11).

**Supplementary Table 3.** Main and interaction effects of two-way non-parametric ANOVA on serum concentration of cytokines, chemokines, and myokines.

| Analysis | Genotype effect | Running effect | Interaction effect |
| --- | --- | --- | --- |
| BDNF^a^ | 1.92 (p=0.17 [p=0.50]) | 2.18 (p=0.14 [p=4.62]) | 0.15 (p=0.70 [p=1.15]) |
| FGF21^a^ | 0.19 (p=0.66 [p=0.76]) | 0.88 (p=0.35 [p=1.05]) | 1.00 (p=0.32 [p=1.50]) |
| FSTL1^a^ | 0.89 (p=0.34 [p=0.60]) | 1.13 (p=0.29 [p=1.90]) | 0.98 (p=0.32 [p=1.33]) |
| Myostatin^a^ | 5.66 (p=0.02 [p=0.14]) | 0.09 (p=0.77 [p=1.02]) | 0.05 (p=0.82 [p=1.04]) |
| Irisin^a^ | 0.58 (p=0.45 [p=0.57]) | 0.23 (p=0.63 [p=0.99]) | 2.52 (p=0.11 [p=3.72]) |
| Oncostatin 5^a^ | 4.87 (p=0.03 [p=0.15]) | 0.50 (p=0.48 [p=1.13]) | 0.58 (p=0.45 [p=1.05]) |
| Osteocrin Musclin^a^ | 14.77 (p=0.0001 [p=0.004]) | 0.23 (p=0.63 [p=1.04]) | 0.06 (p=0.81 [p=1.07]) |
| GM-CSF^b^ | 1.50 (p=0.22 [p=0.43]) | 0.36 (p=0.55 [p=1.01]) | 1.63 (p=0.20 [p=1.33]) |
| IFN-gamma^b^ | 0.10 (p=0.75 [p=0.83]) | 0.49 (p=0.49 [p=1.07]) | 0.74 (p=0.39 [p=1.29]) |
| IL-1alpha^b^ | 2.04 (p=0.15 [p=0.56]) | 0.01 (p=0.99 [p=0.99]) | 0.01 (p=0.98 [p=1.01]) |
| IL-1beta^b^ | 1.79 (p=0.18 [p=0.50]) | 0.01 (p=0.92 [p=1.01]) | 0.10 (p=0.76 [p=1.08]) |
| IL-10^b^ | 1.77 (p=0.18 [p=0.47]) | 0.47 (p=0.49 [p=1.02]) | 0.02 (p=0.89 [p=1.09]) |
| IL-12p70^b^ | 0.81 (p=0.37 [p=0.55]) | 0.92 (p=0.34 [p=1.39]) | 0.72 (p=0.39 [p=1.09]) |
| IL-13^b^ | 0.08 (p=0.78 [p=0.83]) | 0.04 (p=0.85 [p=1.00]) | 1.86 (p=0.17 [p=1.42]) |
| IL-15^b^ | 1.99 (p=0.16 [p=0.52]) | 0.01 (p=0.91 [p=1.03]) | 0.80 (p=0.37 [p=1.37]) |
| IL-17^b^ | 0.20 (p=0.66 [p=0.77]) | 0.98 (p=0.32 [p=1.77]) | 0.08 (p=0.78 [p=1.07]) |
| IL-18^b^ | 1.59 (p=0.21 [p=0.43]) | 1.33 (p=0.25 [p=2.04]) | 0.35 (p=0.55 [p=1.02]) |
| IL-2^b^ | 1.66 (p=0.20 [p=0.46]) | 0.01 (p=0.98 [p=1.01]) | 0.01 (p=0.98 [p=1.08]) |
| IL-22^b^ | 0.25 (p=0.62 [p=0.75]) | 0.07 (p=0.79 [p=1.00]) | 0.13 (p=0.72 [p=1.08]) |
| IL-27^b^ | 0.66 (p=0.42 [p=0.57]) | 0.77 (p=0.38 [p=1.05]) | 0.01 (p=0.99 [p=0.99]) |
| IL-28^b^ | 0.06 (p=0.81 [p=0.84]) | 0.30 (p=0.59 [p=1.02]) | 0.41 (p=0.52 [p=1.08]) |
| IL-3^b^ | 0.01 (p=0.91 [p=0.91]) | 1.65 (p=0.20 [p=2.19]) | 0.01 (p=0.98 [p=1.11]) |
| IL-31^b^ | 0.81 (p=0.37 [p=0.58]) | 0.92 (p=0.34 [p=1.11]) | 0.72 (p=0.39 [p=1.00]) |
| IL-5^b^ | 4.47 (p=0.03 [p=0.16]) | 0.09 (p=0.76 [p=1.05]) | 0.19 (p=0.66 [p=1.15]) |
| IL-6^b^ | 2.51 (p=0.11 [p=0.47]) | 0.06 (p=0.80 [p=0.98]) | 0.36 (p=0.55 [p=1.07]) |
| IL-9^b^ | 1.41 (p=0.23 [p=0.43]) | 0.10 (p=0.75 [p=1.08]) | 0.14 (p=0.71 [p=1.11]) |
| LIF^b^ | 0.73 (p=0.39 [p=0.57]) | 0.15 (p=0.70 [p=1.05]) | 2.08 (p=0.15 [p=1.64]) |
| MCP-1^b^ | 6.73 (p=0.01 [p=0.16]) | 0.96 (p=0.33 [p=1.54]) | 0.01 (p=0.98 [p=1.04]) |
| MCP-3^b^ | 5.04 (p=0.02 [p=0.16]) | 0.01 (p=0.97 [p=1.03]) | 2.26 (p=0.13 [p=2.19]) |
| MSCF^b^ | 0.81 (p=0.37 [p=0.61]) | 0.92 (p=0.34 [p=1.24]) | 0.72 (p=0.39 [p=1.18]) |
| MIP-1alpha^b^ | 1.63 (p=0.20 [p=0.42]) | 0.41 (p=0.52 [p=1.01]) | 0.49 (p=0.49 [p=1.07]) |
| MIP-1beta^b^ | 5.80 (p=0.02 [p=0.18]) | 0.74 (p=0.39 [p=0.99]) | 0.01 (p=0.91 [p=1.08]) |
| TNF-alpha^b^ | 0.20 (p=0.43 [p=0.57]) | 1.89 (p=0.17 [p=2.79]) | 1.49 (p=0.22 [p=1.22]) |

^a^ Data presented as F(1,32) with the corresponding p-value in parenthesis and FDR-adjusted p-value in brackets (n=7-10). ^b^ Data presented as F(1,34) with the corresponding p-value in parenthesis and FDR-adjusted p-value in brackets (n=7-11).

**Supplementary Table 4.** Kendall rank correlation of running distance to protein concentration for wildtype and transgenic animals.

| Analysis | WT | | | TG | | |
| --- | --- | --- | --- | --- | --- | --- |
|  | Kendall tau | p-value | FDR-adjusted p-value | Kendall tau | p-value | FDR-adjusted p-value |
| BDNF^a^ | -0.23 | 0.20 | 0.82 | -0.06 | 0.80 | 0.87 |
| FGF21^a^ | -0.31 | 0.07 | 0.82 | -0.18 | 0.36 | 0.84 |
| FSTL1^a^ | 0.23 | 0.24 | 0.82 | NA | NA | NA |
| Myostatin^a^ | 0.02 | 0.92 | 0.92 | -0.12 | 0.59 | 0.84 |
| Irisin^a^ | -0.19 | 0.34 | 0.82 | 0.12 | 0.59 | 0.84 |
| Oncostatin 5^a^ | NA | NA | NA | 0.17 | 0.42 | 0.84 |
| Osteocrin Musclin^a^ | 0.15 | 0.38 | 0.82 | 0.06 | 0.73 | 0.87 |
| Osteonectin^a^ | -0.11 | 0.52 | 0.82 | 0.11 | 0.55 | 0.84 |
| ENA-78^b^ | 0.35 | 0.04 | 0.82 | 0.29 | 0.12 | 0.77 |
| Eotaxin^b^ | 0.21 | 0.21 | 0.82 | 0.53 | 0.00 | 0.17 |
| G-CSF^b^ | -0.07 | 0.68 | 0.82 | -0.26 | 0.17 | 0.77 |
| GM-CSF^b^ | -0.24 | 0.22 | 0.82 | 0.14 | 0.53 | 0.84 |
| GRO-alpha^b^ | -0.10 | 0.54 | 0.82 | 0.10 | 0.61 | 0.84 |
| IFN-gamma^b^ | -0.24 | 0.20 | 0.82 | 0.11 | 0.60 | 0.84 |
| IL-1alpha^b^ | -0.04 | 0.80 | 0.86 | -0.01 | 0.96 | 0.96 |
| IL-1beta^b^ | -0.12 | 0.51 | 0.82 | 0.10 | 0.65 | 0.84 |
| IL-10^b^ | -0.16 | 0.35 | 0.82 | -0.12 | 0.54 | 0.84 |
| IL-12p70^b^ | 0.07 | 0.73 | 0.82 | NA | NA | NA |
| IL-13^b^ | -0.19 | 0.34 | 0.82 | 0.30 | 0.17 | 0.77 |
| IL-15^b^ | -0.15 | 0.44 | 0.82 | 0.14 | 0.53 | 0.84 |
| IL-17^b^ | -0.21 | 0.28 | 0.82 | -0.05 | 0.80 | 0.87 |
| IL-18^b^ | 0.15 | 0.39 | 0.82 | 0.10 | 0.58 | 0.84 |
| IL-2^b^ | -0.07 | 0.70 | 0.82 | NA | NA | NA |
| IL-22^b^ | -0.02 | 0.92 | 0.92 | -0.08 | 0.67 | 0.84 |
| IL-23^b^ | -0.10 | 0.54 | 0.82 | -0.28 | 0.13 | 0.77 |
| IL-27^b^ | -0.19 | 0.26 | 0.82 | -0.20 | 0.30 | 0.84 |
| IL-28^b^ | -0.08 | 0.66 | 0.82 | -0.19 | 0.36 | 0.84 |
| IL-3^b^ | 0.07 | 0.73 | 0.82 | 0.19 | 0.40 | 0.84 |
| IL-31^b^ | 0.07 | 0.73 | 0.82 | NA | NA | NA |
| IL-4^b^ | 0.04 | 0.80 | 0.86 | 0.24 | 0.21 | 0.77 |
| IL-5^b^ | -0.18 | 0.29 | 0.82 | 0.04 | 0.83 | 0.87 |
| IL-6^b^ | -0.10 | 0.59 | 0.82 | 0.04 | 0.85 | 0.87 |
| IL-9^b^ | -0.03 | 0.86 | 0.91 | -0.09 | 0.68 | 0.84 |
| IP-10^b^ | 0.12 | 0.46 | 0.82 | 0.24 | 0.20 | 0.77 |
| LIF^b^ | 0.06 | 0.70 | 0.82 | -0.22 | 0.23 | 0.77 |
| MCP-1^b^ | -0.08 | 0.63 | 0.82 | -0.04 | 0.85 | 0.87 |
| MCP-3^b^ | -0.18 | 0.29 | 0.82 | 0.32 | 0.09 | 0.77 |
| MSCF^b^ | 0.07 | 0.73 | 0.82 | NA | NA | NA |
| MIP-1alpha^b^ | 0.07 | 0.72 | 0.82 | 0.30 | 0.17 | 0.77 |
| MIP-1beta^b^ | 0.07 | 0.68 | 0.82 | 0.11 | 0.57 | 0.84 |
| MIP-2^b^ | 0.19 | 0.26 | 0.82 | 0.08 | 0.67 | 0.84 |
| RANTES^b^ | 0.19 | 0.26 | 0.82 | 0.27 | 0.14 | 0.77 |
| TNF-alpha^b^ | 0.19 | 0.34 | 0.82 | NA | NA | NA |

^a^ n=7-10, ^b^ n=7-11

**Supplementary Table 5.** Linear correlation of running distance (km) to protein concentration (pg/ml) in normally distributed analyte data from Supplementary Table 1.

| Analysis | WT | | | TG | | |
| --- | --- | --- | --- | --- | --- | --- |
|  | Pearson r | p-value | FDR-adjusted p-value | Pearson r | p-value | FDR-adjusted p-value |
| Osteonectin^b^ | -0.30 | 0.43 | 0.61 | -0.51 | 0.13 | 0.33 |
| IL-4^c^ | -0.51 | 0.16 | 0.52 | 0.07 | 0.85 | 1.06 |
| MIP2^c^ | 0.06 | 0.88 | 0.88 | 0.02 | 0.97 | 1.07 |
| IL-23^a,^^c^ | -0.37 | 0.33 | 0.54 | -0.53 | 0.11 | 0.37 |
| RANTES^a,c^ | -0.18 | 0.64 | 0.72 | 0.46 | 0.19 | 0.37 |
| IP-10^a,c^ | -0.54 | 0.14 | 0.68 | 0.44 | 0.21 | 0.35 |
| ENA-78^a,c^ | 0.48 | 0.19 | 0.38 | 0.70 | 0.02 | 0.12 |
| GRO-alpha^a,c^ | -0.50 | 0.17 | 0.43 | 0.00 | 1.0 | 1.00 |
| Eotaxin^a,c^ | 0.26 | 0.50 | 0.63 | 0.75 | 0.01 | 0.13 |
| G-CSF^a,c^ | -0.61 | 0.08 | 0.81 | -0.17 | 0.64 | 0.91 |

^a^ Log transformed data

^b^ n=9-10

^c^ n=10-11
